# Molecular Basis of Gene-Gene Interaction: Cyclic Cross-Regulation of Gene Expression and Post-GWAS Gene-Gene Interaction Involved in Atrial Fibrillation

**DOI:** 10.1371/journal.pgen.1005393

**Published:** 2015-08-12

**Authors:** Yufeng Huang, Chuchu Wang, Yufeng Yao, Xiaoyu Zuo, Shanshan Chen, Chengqi Xu, Hongfu Zhang, Qiulun Lu, Le Chang, Fan Wang, Pengxia Wang, Rongfeng Zhang, Zhenkun Hu, Qixue Song, Xiaowei Yang, Cong Li, Sisi Li, Yuanyuan Zhao, Qin Yang, Dan Yin, Xiaojing Wang, Wenxia Si, Xiuchun Li, Xin Xiong, Dan Wang, Yuan Huang, Chunyan Luo, Jia Li, Jingjing Wang, Jing Chen, Longfei Wang, Li Wang, Meng Han, Jian Ye, Feifei Chen, Jingqiu Liu, Ying Liu, Gang Wu, Bo Yang, Xiang Cheng, Yuhua Liao, Yanxia Wu, Tie Ke, Qiuyun Chen, Xin Tu, Robert Elston, Shaoqi Rao, Yanzong Yang, Yunlong Xia, Qing K. Wang

**Affiliations:** 1 Key Laboratory of Molecular Biophysics of the Ministry of Education, Cardio-X Center, College of Life Science and Technology and Center for Human Genome Research, Huazhong University of Science and Technology, Wuhan, China; 2 Sun Yat-sen University Cancer Center, State Key Laboratory of Oncology in South China, Collaborative Innovation Center for Cancer Medicine, Guangzhou, China; 3 Department of Cardiology, First Affiliated Hospital of Dalian Medical University, Dalian, China; 4 Department of Cardiology, People’s Hospital, Wuhan University, Wuhan, China; 5 Department of Cardiology, Union Hospital, Huazhong University of Science and Technology, Wuhan, China; 6 Department of Cardiology, the First Affiliated Hospital of Wuhan City, Wuhan, China; 7 Center for Cardiovascular Genetics, Department of Molecular Cardiology, Lerner Research Institute, Cleveland Clinic, Cleveland, Ohio, United States of America; 8 Department of Molecular Medicine, Department of Genetics and Genome Sciences, Case Western Reserve University, Cleveland, Ohio, United States of America; 9 Department of Epidemiology and Biostatistics, Case Western Reserve University School of Medicine, Cleveland, Ohio, United States of America; 10 Institute of Medical Systems Biology and Department of Medical Statistics and Epidemiology, School of Public Health, Guangdong Medical College, Dongguan, China; Dartmouth College, UNITED STATES

## Abstract

Atrial fibrillation (AF) is the most common cardiac arrhythmia at the clinic. Recent GWAS identified several variants associated with AF, but they account for <10% of heritability. Gene-gene interaction is assumed to account for a significant portion of missing heritability. Among GWAS loci for AF, only three were replicated in the Chinese Han population, including SNP rs2106261 (G/A substitution) in *ZFHX3*, rs2200733 (C/T substitution) near *PITX2c*, and rs3807989 (A/G substitution) in *CAV1*. Thus, we analyzed the interaction among these three AF loci. We demonstrated significant interaction between rs2106261 and rs2200733 in three independent populations and combined population with 2,020 cases/5,315 controls. Compared to non-risk genotype GGCC, two-locus risk genotype AATT showed the highest odds ratio in three independent populations and the combined population (OR=5.36 (95% CI 3.87-7.43), P=8.00×10^-24^). The OR of 5.36 for AATT was significantly higher than the combined OR of 3.31 for both GGTT and AACC, suggesting a synergistic interaction between rs2106261 and rs2200733. Relative excess risk due to interaction (RERI) analysis also revealed significant interaction between rs2106261 and rs2200733 when exposed two copies of risk alleles (RERI=2.87, *P<1.00*×10^-4^) or exposed to one additional copy of risk allele (RERI=1.29, *P<1.00*×10^-4^). The INTERSNP program identified significant genotypic interaction between rs2106261 and rs2200733 under an additive by additive model (OR=0.85, 95% CI: 0.74-0.97, *P*=0.02). Mechanistically, *PITX2c* negatively regulates expression of *miR-1*, which negatively regulates expression of *ZFHX3*, resulting in a positive regulation of *ZFHX3* by *PITX2c*; *ZFHX3* positively regulates expression of *PITX2C*, resulting in a cyclic loop of cross-regulation between *ZFHX3* and *PITX2c*. Both *ZFHX3* and *PITX2c* regulate expression of *NPPA*, *TBX5* and *NKX2*.*5*. These results suggest that cyclic cross-regulation of gene expression is a molecular basis for gene-gene interactions involved in genetics of complex disease traits.

## Author Summary

Gene-gene interaction is assumed to be critical to the pathogenesis of human disease, but its contribution to human disease phenotype needs definitive documentation. Moreover, the underlying molecular mechanism for gene-gene interaction is unknown. Here we use atrial fibrillation (AF) as a model to demonstrate that gene-gene interaction plays an important role in disease pathogenesis. Only three of the ten AF loci identified by GWAS in European ancestry populations, including *PITX2c*, *ZFHX3*, and *CAV1*, were replicated in the Chinese population and thus selected for gene-gene interaction studies. We show that the *PITX2c* locus interacts with the *ZHFX3* locus to increase the risk of AF. Because gene-gene interaction can generate synergistic effect that markedly increases risk of AF, we conclude that gene-gene interaction accounts for a significant portion of heritability of AF. Mechanistically, *PITX2c* positively regulates *ZHFX3 via miR-1* and *ZHFX3* positively regulates *PITX2c*, which generates a loop of cross-regulation of the two genes. Our study suggests that cyclic cross-regulation of gene expression is a molecular basis for gene-gene interaction involved in disease phenotype.

## Introduction

Genome-wide association studies (GWAS) have been highly successful in identifying common genomic variants that are associated with complex human diseases or traits. However, these common variants have small effects, and in aggregate explain only a small fraction of heritability for most diseases or traits. The major portion of heritability remains missing, and this represents a major dilemma in complex trait genetics referred to as “missing heritability”. Gene-gene interaction has been proposed to be a contributor to the problem of missing heritability.

Gene-gene interaction has been long known to have an impact on an organism’s phenotype, for example, the color of a flower in plants and the color of a fly’s eye. However, it has been challenging to detect gene-gene interaction in human GWAS. Moreover, no gene interaction was functionally validated. Considering the potentially large number of gene-gene interaction, identification of true and casual interaction has been proven to be a daunting task. However, without doubt, studies of gene-gene interaction will contribute to the understanding of inheritance, particularly inheritance of important diseases and traits, and provide insights into the biological pathways and molecular mechanisms of disease pathogenesis.

Atrial fibrillation (AF) is the most common cardiac arrhythmia seen at the clinical setting and accounts for approximately one-third of hospitalizations for cardiac rhythm disturbances [1]. The prevalence of AF is 0.4%-1.0% in the general population, and increases with age, reaching 8% in people over 80 [1]. A similar prevalence rate of 0.77% was found for AF in the Chinese population [2]. AF accounts for 15% of all strokes, worsens heart failure, and independently increases the risk of stroke 5-fold and risk of cardiac death up to 1.9-fold [3]. Genetic factors play an important role in the pathogenesis of AF. The heritability of polygenic liability to AF has been estimated to be 0.62 [4].

To date, several major GWAS have been reported for common complex AF and identified variants in ten chromosomal loci that were associated with AF. The first GWAS for AF identified significant association between SNP rs2200733 near the *PITX2c* gene encoding paired-like homeodomain 2 transcript c on chromosome 4q25 and AF in several populations of European ancestry as well as one Hong Kong population [5]. Our group later reported that SNP rs2200733 confers a significant risk in the mainland Chinese Han population, too [6]. Then, two independent GWAS identified significant association between AF and SNPs rs2106261 [7] and rs7193343 [8], both of which are located in the *ZFHX3* gene encoding zinc finger homeobox 3 on chromosome 16q22. We have found that rs2106261, but not rs7193343, showed significant association with AF in the Chinese Han population [9]. Later, a common variant in *KCNN3* (encoding potassium intermediate/small conductance calcium-activated channel, subfamily N, member 3), rs13376333, was found to be associated with lone AF [10]. However, we found that rs13376333 did not show significant association with AF in the Chinese Han population [9]. Ellinor et al [11] identified six susceptibility loci for AF through meta-GWAS analysis. We have shown that only one SNP, rs3807989 at the *CAV1* locus (encoding caveolin 1) among the six loci, were associated with AF in the Chinese Han population [12]. In this study, we studied the gene-gene interaction for three AF loci replicated in the Chinese population, i.e. SNP rs2106261 in *ZFHX3*, rs2200733 near *PITX2c*, and rs3807989 in *CAV1*. We provide strong genetic evidence that SNP rs2200733 near *PITX2c* and rs2106261 in *ZFHX3* interact with each other, resulting in a synergistic effect that increases the odds ratios (ORs) to risk of AF. Most importantly, we also carried out a series of cellular and molecular studies to identify the molecular mechanisms underlying the gene–gene interaction. We found that *PITX2c* and *ZFHX3* cross-regulate each other’s expression as well as expression of downstream genes such as *NPPA* (encoding atrial natriuretic factor or ANF), providing a novel molecular basis for their interaction at the molecular genetic level.

## Results

### Significant association between SNP rs2200733 near *PITX2c* on 4q25 and rs2106261 in *ZFHX3* on 16q22 and AF in three independent populations

We previously reported that among the first three genetic loci for AF identified by GWAS in European ancestry populations, only rs2200733 at the *PITX2c* locus on 4q25 and rs2106261 in *ZFHX3* on 16q22, but not rs13376333 in *KCNN3*, were replicated in the Chinese Han populations [6,9]. We, therefore, carried out a deeper study to determine whether there is gene-gene interaction between rs2200733 and rs2106261. We utilized a case control design which involves three independent populations. The initial association study was carried out with 569 AF patients and 1,996 non-AF control samples (referred to as the Discovery population). The positive findings in the Discovery population were validated in two independent replication populations. The first replication population consisted of 641 AF cases and 1,692 controls (referred to as Replication I population). The second replication population consisted of 810 cases and 1,627 controls (referred to as Replication II population). The clinical characteristics of the three study populations are shown in S1 Table.

We first examined the association of AF with each GWAS SNP individually. There was no deviation from the Hardy-Weinberg equilibrium for the two SNPs, rs2200733 and rs2106261 in the control groups of the three populations (S2 Table).

As shown in S3 Table, SNP rs2200733 showed highly significant association with AF in the Discovery population with a *P* value of 1.58×10^-14^ (OR = 1.70 (95% CI 1.48–1.94)) with the T allele as the risk allele. After adjusting for covariates of age and gender with multivariable logistical regression analysis, rs2200733 remained significantly associated with AF (*Padj* = 5.50×10^-13^, OR = 1.32 (95% CI 1.22–1.42)). SNP rs2200733 remained significant association with AF in Replication I population (*Pobs* = 1.27×^10-11^, OR = 1.57 (95% CI 1.38–1.79); *Padj* = 3.17×10^-10^, OR = 1.27 (95% CI 1.18–1.37)) and Replication II population (*Pobs* = 2.20×10^-10^, OR = 1.48 (95% CI 1.31–1.67); *Padj* = 7.84×10^-10^, OR = 1.22 (95% CI 1.14–1.29)). In the combined population, the association between SNP rs2200733 and AF was highly significant (*Pobs* = 2.83×10^-33^, OR = 1.57 (95% CI 1.46–1.69); *Padj* = 4.54×10^-29^, OR = 1.26 (95% CI 1.21–1.31)). In addition to analysis of allelic association, we also analyzed genotypic association assuming three different genetic models. As shown in S4 Table, highly significant genotypic associations were detected between SNP rs2200733 and AF in the Discovery population, Replication I population and Replication II population in an additive, dominant, or recessive model. In the combined cohort of the three populations, the genotypic associations between SNP rs2200733 and AF were also highly significant with *Padj* of 4.54×10^-29^ (OR = 1.61 (95% CI 1.48–1.75)), 1.82×10^-23^ (OR = 1.36 (95% CI 1.28–1.45)) and 4.69×10^-16^ (OR = 1.37 (95% CI 1.27–1.48)) under an additive, recessive and dominant model, respectively (S4 Table).

Similarly, SNP rs2106261 on 16q22 also showed significant allelic and genotypic association with AF in the Discovery population, Replication I population and Replication II population (S3 and S4 Tables, respectively). In the combined population, the allelic association between SNP rs2106261 and AF was highly significant (*Pobs* = 6.26×10^-12^, OR = 1.30 (95% CI 1.21–1.40); *Padj* = 3.03×10^-12^, OR = 1.16 (95% CI 1.11–1.21)) (S3 Table) with the A allele as the risk allele. Genotypic associations were also identified between rs2106261 and AF (*Padj* of 3.11×10^-12^ (OR = 1.33 (95% CI 1.23–1.45)), 1.02×10^-12^ (OR = 1.35 (95% CI 1.24–1.46)) and 1.42×10^-6^ (OR = 1.15 (95% CI 1.08–1.21)) under an additive, recessive and dominant model, respectively (S4 Table).

### Gene-gene interaction between *ZFHX3* variant rs2106261 and *PITX2c* variant rs2200733

**Fig 1:**
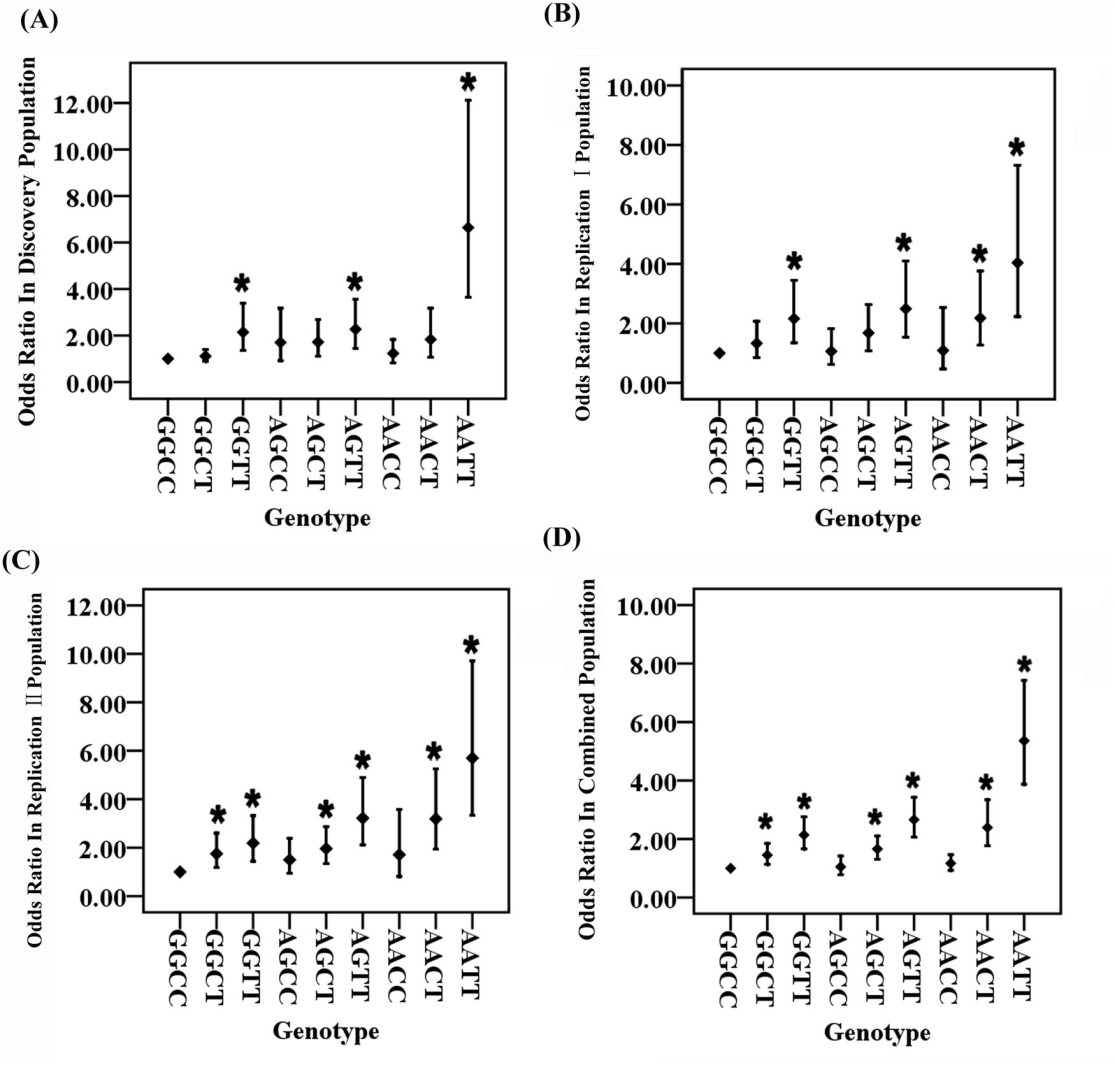
Odds ratios (ORs) for each two-locus genotype for GWAS SNPs rs2106261 in the *ZFHX3* gene and rs2200733 close to the *PITX2c* gene involved in the pathogenesis of AF after adjustment for covariates. For two SNPs, there are a total of 9 genotypes. The wild type or non-risk GGCC genotype was used as the reference and ORs for other genotypes were estimated against the reference genotype using multivariable logistic regression analysis by including the age and gender as covariates. A. Analysis of ORs in the Discovery population. B. Analysis of ORs in the Replication I population. C. Analysis of ORs in the Replication II population. D. Analysis of ORs in the combined population with the Discovery, Replication I and Replication II cohorts. *P<0.01.

To study the interaction between rs2106261 (G to A substitution, risk allele = A) and rs2200733 (C to T substitution, risk allele = T), we first defined the frequencies of nine possible two-locus genotypes (3^2^ genotypes: GGCC, GGCT, GGTT, AGCC, AGCT, AGTT, AACC, AACT, AATT) in cases and controls of the three independent study populations. Then, we used the wild type non-risk GGCC genotype (non-risk homozygote for both loci) as baseline or reference, and estimated the OR for each of the eight other genotypes. As shown in Table 1 and Fig 1 and S1 Fig, compared with the GGCC non-risk reference genotype, the double risk homozygous genotype AATT showed a dramatically increased risk for AF with the highest ORs of 4.81 (95% CI 2.88–8.04) (*Pobs* = 3.83×10^-10^) and 6.64 (95% CI 3.64–12.11) (*Padj* = 6.38×10^-10^) before and after adjustment for covariates of age and gender, respectively, in the Discovery population. This interesting finding was replicated in two independent AF populations with ORs of 4.04 (95% CI 2.23–7.32) (*Padj* = 4.34×10^-6^, Replication I) and 5.70 (95% CI 3.34–9.71) (*Padj* = 1.58×10^-10^, Replication II). In the combined cohort of the three populations, AATT increased risk of AF with an OR of 5.36 (95% CI 3.87–7.43) (*Padj* = 8.00×10^-24^) (Table 1).

**Table 1:** ORs for 8 two-locus genotypes versus non-risk homozygous genotype GGCC as a reference for SNPs rs2200733 and rs2106261 in the Chinese Han populations.

Two-locus genotype				Allele test^a^		Adjust for age and gender^b^	
rs2106261	rs2200733	N of cases (%)	N of controls (%)	*P* value	OR (95%CI)	*P* value	OR (95%CI)
Discovery population 569 cases /1,996 controls							
GG	CC	44 (7.7%)	230 (11.5%)	N.A‡	1.00	N.A	1.00
GG	CT	106 (18.6%)	475 (23.8%)	0.43	1.17 (0.79–1.72)	0.34	1.11 (0.89–1.39)
GG	TT	83 (14.6%)	206 (10.3%)	3.27×10^-4^	2.11 (1.40–3.18)	1.0×10^-3^	2.14 (1.36–3.38)
AG	CC	21 (3.7%)	206 (10.3%)	0.02	1.88 (1.08–3.26)	0.10	1.70 (0.92–3.17)
AG	CT	117 (20.6%)	420 (21.0%)	0.05	1.46 (0.99–2.13)	0.02	1.72 (1.11–2.68)
AG	TT	99 (17.4%)	243 (12.2%)	1.66×10^-4^	2.13 (1.43–3.17)	1.66×10^-4^	2.27 (1.45–3.56)
AA	CC	13 (2.3%)	51 (2.6%)	0.41	1.33 (0.67–2.65)	0.31	1.23 (0.83–1.83)
AA	CT	40 (7.0%)	115 (5.8%)	0.02	1.82 (1.12–2.95)	0.03	1.83 (1.06–3.17)
AA	TT	46 (8.1%)	50 (2.5%)	3.83×10^-10^	4.81 (2.88–8.04)	6.38×10^-10^	6.64 (3.64–12.11)
Replication I population 641 cases /1,692 controls							
GG	CC	43 (6.7%)	199 (11.8%)	N.A	1.00	N.A	1.00
GG	CT	129 (20.1%)	413 (24.4%)	0.06	1.45 (0.98–2.12)	0.22	1.33 (0.85–2.07)
GG	TT	87 (13.6%)	176 (10.4%)	8.44×10^-5^	2.29 (1.51–3.47)	1.00×10^-3^	2.16 (1.35–3.45)
AG	CC	44 (6.9%)	166 (9.8%)	0.39	1.23 (0.77–1.96)	0.83	1.06 (0.62–1.82)
AG	CT	139 (21.7%)	378 (22.3%)	6.00×10^-3^	1.70 (1.16–2.50)	0.02	1.68 (1.08–2.63)
AG	TT	96 (15.0%)	163 (9.6%)	1.43×10^-6^	2.73 (1.80–4.13)	1.80×10^-4^	2.49 (1.54–4.10)
AA	CC	10 (1.6%)	47 (2.8%)	0.97	1.02 (0.48–2.17)	0.85	1.09(0.47–2.53)
AA	CT	47 (7.3%)	104 (6.1%)	2.00×10^-3^	2.09 (1.30–3.37)	5.00×10^-3^	2.18 (1.27–3.76)
AA	TT	46 (7.2%)	46 (2.7%)	2.65×10^-9^	4.63 (2.74–7.82)	4.34×10^-6^	4.04 (2.23–7.32)
Replication II population 810 cases /1,627 controls							
GG	CC	44 (5.4%)	176 (10.8%)	N.A	1.00	N.A	1.00
GG	CT	161 (19.9%)	379 (23.3%)	6.00×10^-3^	1.70 (1.16–2.48)	6.00×10^-3^	1.75 (1.18–2.60)
GG	TT	108 (13.3%)	197 (12.1%)	1.22×10^-4^	2.19 (1.46–3.29)	2.25×10^-4^	2.19 (1.44–3.32)
AG	CC	56 (6.9%)	162 (10.0%)	0.16	1.38 (0.88–2.17)	0.09	1.50 (0.95–2.39)
AG	CT	189 (23.3%)	404 (24.8%)	1.00×10^-3^	1.87 (1.29–2.72)	1.00×10^-3^	1.96 (1.34–2.87)
AG	TT	124 (15.3%)	160 (9.8%)	2.29×10^-8^	3.10 (2.07–4.65)	4.36×10^-8^	3.22 (2.12–4.90)
AA	CC	15 (1.9%)	30 (1.8%)	0.05	2.00 (0.99–4.04)	0.16	1.71 (0.81–3.58)
AA	CT	57 (7.0%)	74 (4.5%)	2.53×10^-6^	3.08 (1.91–4.97)	4.66×10^-6^	3.19 (1.94–5.25)
AA	TT	56 (6.9%)	45 (2.8%)	1.92×10^-10^	4.98 (2.98–8.31)	1.58×10^-10^	5.70 (3.34–9.71)
Combined population 2,020 cases /5,315 controls							
GG	CC	131 (6.5%)	605 (11.4%)	N.A	1.00	N.A	1.00
GG	CT	396 (19.6%)	1,267 (23.8%)	1.00×10^-3^	1.44 (1.16–1.80)	3.00×10^-3^	1.45 (1.13–1.85)
GG	TT	278 (13.8%)	579 (10.9%)	2.58×10^-11^	2.22 (1.75–2.81)	5.47×10^-9^	2.14 (1.66–2.76)
AG	CC	121 (6.0%)	534 (10.0%)	0.74	1.05 (0.80–1.38)	0.74	1.05 (0.78–1.42)
AG	CT	445 (22.0%)	1,202 (22.6%)	1.19×10^-6^	1.71 (1.38–2.13)	2.49×10^-5^	1.66 (1.31–2.11)
AG	TT	319 (15.8%)	566 (10.6%)	3.15×10^-16^	2.60 (2.06–3.29)	8.15×10^-14^	2.66 (2.06–3.43)
AA	CC	38 (1.9%)	128 (2.4%)	0.13	1.37 (0.91–2.06)	0.18	1.17 (0.93–1.46)
AA	CT	144 (7.1%)	293 (5.5%)	3.17×10^-9^	2.27 (1.73–2.99)	1.56×10^-8^	2.39 (1.77–3.34)
AA	TT	148 (7.3%)	141 (2.7%)	2.96×10^-27^	4.85 (3.60–6.53)	8.00×10^-24^	5.36 (3.87–7.43)

OR: odds ratio; CI: confidence interval

^a^nominal *P* value and OR computed using Chi-square tests with Pearson’s 2×2 contingency tables.

^b^nominal *P* value and OR computed using multivariable logistic regression analysis including age and gender as covariates.

The ORs among the different genotypes were compared for statistical significance using the Breslow-Day test (S5 Table). In all three independent populations as well as the combined population, ORs for genotype AATT (double risk homozygotes for both rs2106261 and rs2200733) were significantly higher than the ORs for each single-risk homozygotes (GGTT, homozygous risk genotype for rs2106261; AACC, homozygous risk genotype for rs2200733) (Table 1 and S5 Table). Moreover, the OR of 6.64 for double-risk homozygote AATT was higher than the combined ORs for the two single-risk homozygotes GGTT and AACC together (2.14+1.25 = 3.39) in the Discovery population (Table 1, Fig 1 and S1 Fig). Similar findings were observed in the Replication I population (4.34 vs. 3.25 (2.16+1.09)), the replication II population (5.70 vs. 3.90 (2.19+1.71)), or the combined cohort (5.36 vs. 3.31 (2.14+1.17)) (Table 1, Fig 1 and S1 Fig). These data provide genetic evidence for interaction between *ZFHX3* variant rs2106261 and *PITX2c* variant rs2200733, which generates a synergistic effect that markedly increases the risk of AF.

Two other genotypes, GGTT and AGTT, significantly increased risk of AF compared to reference non-risk genotype GGCC, consistently in all three populations (*Padj*<0.006 after Bonferroni correction) (Table 1, Fig 1 and S1 Fig).

### Molecular basis of gene-gene interaction: *PITX2c* positively regulates the expression of *ZFHX3* via miR-1

**Fig 2:**
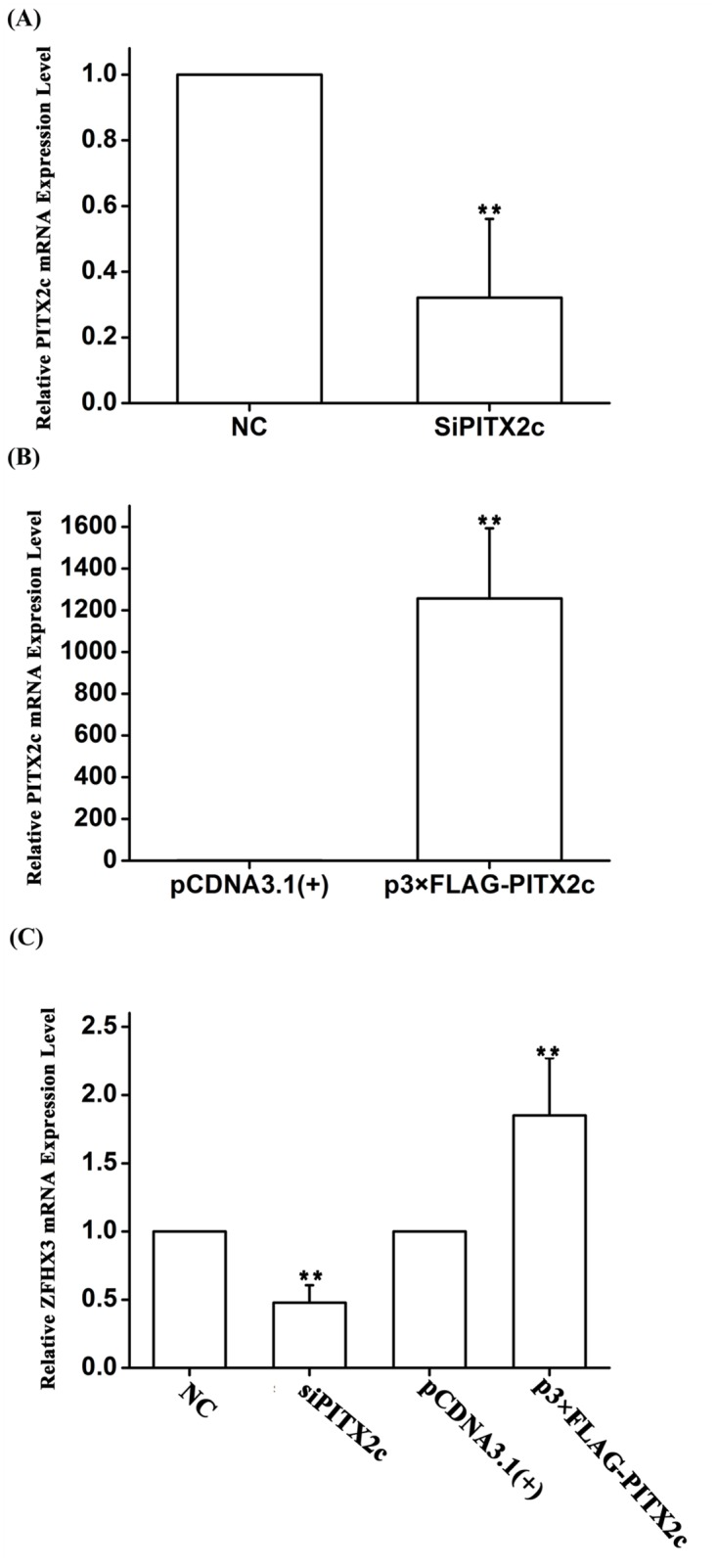
The *PITX2c* gene positively regulates expression of the *ZFHX3* gene. HCT116 cells were transfected with siRNA specific for *PITX2c* or an expression plasmid for *PITX2c* and used for isolation of total RNA samples and real time RT-PCR analysis. A. Real-time RT-PCR analysis for *PITX2c*. Transfection of siRNA for *PITX2c* successfully reduced expression of *PITX2c*. B. Real-time RT-PCR analysis for *PITX2c*. Transfection of an expression plasmid for *PITX2c* successfully increased expression of *PITX2c*. C. Real-time RT-PCR analysis for *ZFHX3*. Transfection of siRNA for *PITX2c* reduced expression of *ZFHX3*. Transfection of an expression plasmid for *PITX2c* successfully increased expression of *ZFHX3*. **P<0.01.

To substantiate the novel finding of the genetic interaction between rs2106261 and rs2200733 as identified by the analyses above, we carried out functional studies to identify the underlying molecular mechanism of the interaction. The *PITX2c* gene near rs2200733 has been demonstrated to be an AF gene using mouse models and shown to regulate several genes in the atria [13–15]. Because *PITX2c* encodes a transcriptional factor, we hypothesized that *PITX2c* would regulate the expression of *ZFHX3*, generating a synergistic effect for gene-gene interaction. To test this hypothesis, we transfected HCT116 cells with a *PITX2c*-specific siRNA and a negative control siRNA (NC control) and then used real-time RT-PCR analysis to measure the expression level of *ZFHX3*. As shown in Fig 2, knockdown of *PITX2c* expression by siRNA significantly decreased the expression level of *ZFHX3* (*P* = 4.00×10^-3^) (Fig 2A and 2C). In a parallel study, overexpression of a FLAG-tagged PITX2c protein by transfection of a p3×FLAG-PITX2c expression plasmid significantly increased the expression level of *ZFHX3* (*P* = 0.01) (Fig 2B and 2C). These studies indicate that *PITX2c* positively regulates expression of *ZFHX3*.

**Fig 3:**
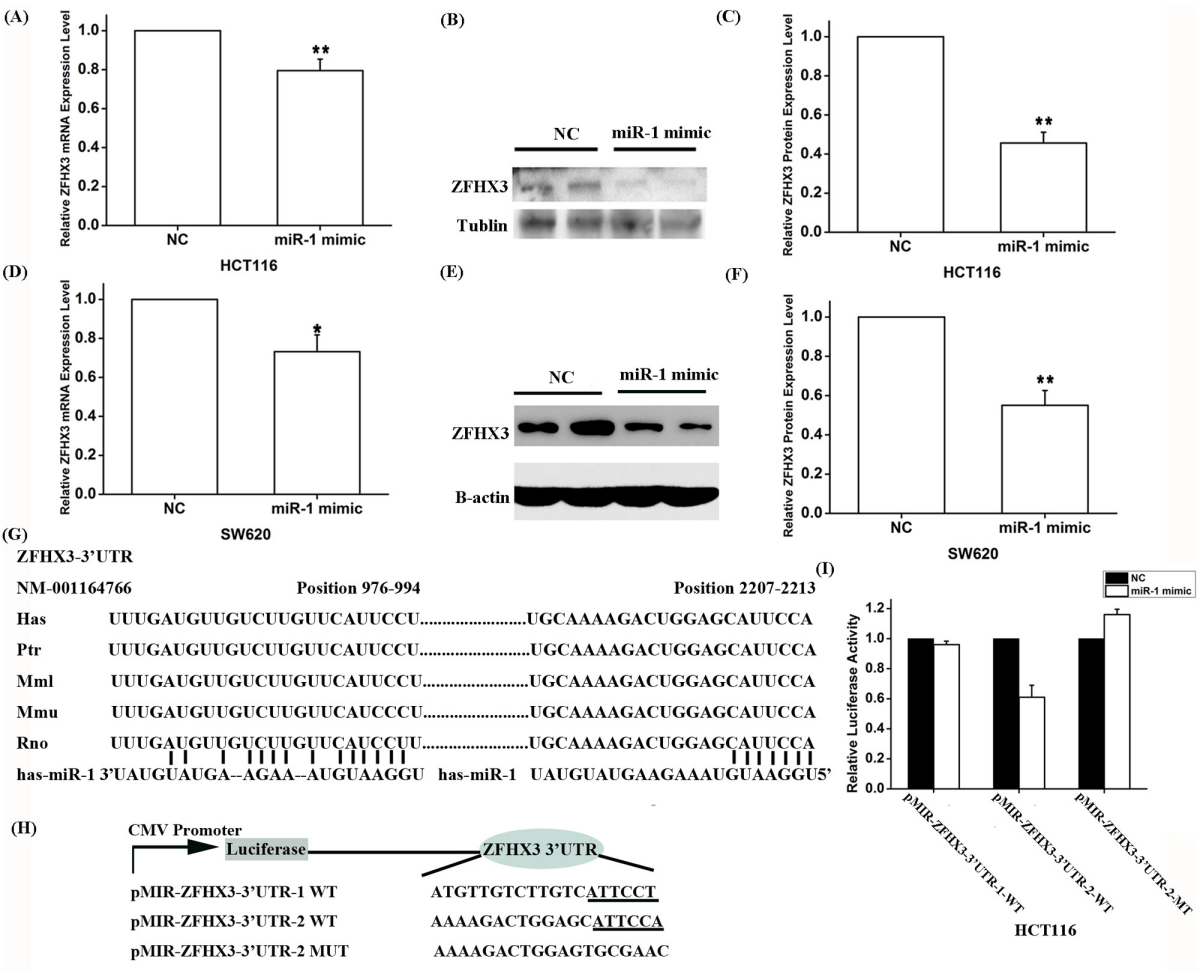
Expression of *ZFHX3* is negatively regulated by *miR-1*. HCT116 (**A-C**) and SW620 (**D-F**) cells were transfected with miR-1 mimics and negative control mimics (NC) and used for isolation of RNA samples for real-time RT-PCR analysis or for isolation of protein extracts for Western blot analysis for the expression levels of *ZFHX3* mRNA and protein. A. Real-time RT-PCR analysis revealed that the *miR-1* mimics reduced the expression of *ZFHX3* by 20% in HCT116 cells (*P* = 0.004). B, C. Western blot analysis revealed that the *miR-1* mimics reduced the expression of the ZFHX3 protein by 54% in HCT116 cells (*P* = 6.84×10^-5^). D. Real-time RT-PCR analysis revealed that the *miR-1* mimics reduced the expression of *ZFHX3* by 27% in SW620 cells (*P* = 0.01). E, F. Western blot analysis revealed that the *miR-1* mimics reduced the expression of the ZFHX3 protein by 45% in SW620 cells (*P* = 4.887×10^-4^). G. Identification of two putative *miR-1* binding sites at the 3’-UTR of *ZFHX3* by bioinformatic analysis and alignment of miR-1 binding sequences across species. H. A schematic diagram shows luciferase reporters containing the potential miR-1 binding site or the related mutated site. I. *MiR-1* targets the second *miR-1* binding site to regulate expression of *ZFHX3*. Luciferase assays revealed that compared to negative control mimics, miR-1 mimics significantly reduced luciferase activities from pMIR-ZFHX3–3’-UTR-2, but not from pMIR-ZFHX3-3’-UTR-1. *P<0.05; **P<0.01.

To explore the molecular mechanism by which *PITX2c* regulates *ZFHX3*, we searched for a potential *PITX2c* binding site at the *ZFHX3* promoter and regulatory region, but failed to find one. Because *PITX2c* was shown to negatively regulate the expression of *miR-1* (microRNA 1–1) [15], we hypothesize that *PITX2c* may regulate expression of *ZFHX3* through *miR-1*. To test this hypothesis, we transfected HCT116 cells with *miR-1* mimics and control microRNA mimics and measured the expression level of *ZFHX3*. Both real-time RT-PCR analysis and Western blot analysis showed that *miR-1* mimics significantly decreased expression of *ZFHX3* at both mRNA (*P* = 4.00×10^-4^) and protein levels (*P* = 6.84×10^-5^), although the effect on the protein level was more robust (Fig 3A–3C). This interesting finding of down-regulation of *ZFHX3* by *miR-1* was confirmed in another cell line, SW620 at the *ZFHX3* mRNA (*P* = 0.01) and protein levels (*P* = 4.89×10^-4^) (Fig 3D–3F). These results suggest that *miR-1* negatively regulates expression of *ZFHX3*.

To explore the molecular mechanism by which *miR-1* regulates *ZFHX3*, we performed bioinformatic analysis by searching two databases, DIANA TOOLs and microRNA.org-Target and Expression, and found that the 3’-untranslated region (3’-UTR) of *ZFHX3* contained two potential targeting sites for *miR-1* (Fig 3G). We cloned each region containing a *miR-1* binding site downstream of the firefly luciferase coding region in the pMIR-REPORT luciferase vector, resulting in luciferase reporters pMIR-ZFHX3-3’-UTR-1 (cloned genomic region: chr16: 72819500 to 72820662) and pMIR-ZFHX3–3’-UTR-2 (cloned genomic region: chr16: 72818241 to 72819390), respectively (Fig 3H). Each reporter was co-transfected with *miR-1* mimics (100 nM) into HCT116 cells and luciferase assays were carried out. A schematic diagram shows luciferase reporters containing the potential *miR-1* binding site or the related mutated site (Fig 3H). As shown in Fig 3I, *miR-1* mimics significantly reduced luciferase activities from pMIR-ZFHX3–3’-UTR-2, but not that from pMIR-ZFHX3–3’-UTR-1. Mutation of the miR-1 binding site in pMIR-ZFHX3–3’-UTR-2 from CATTCCA to TGCGAAC abolished the *miR-1*-mediated reduction of the reporter luciferase activity (Fig 3I). These data suggest that *miR-1* negatively regulates expression of *ZFHX3* by targeting to the second binding site at the 3’-UTR of *ZFHX3*.

### Molecular basis of gene-gene interaction: *ZFHX3* positively regulates the expression of *PITX2c*

**Fig 4:**
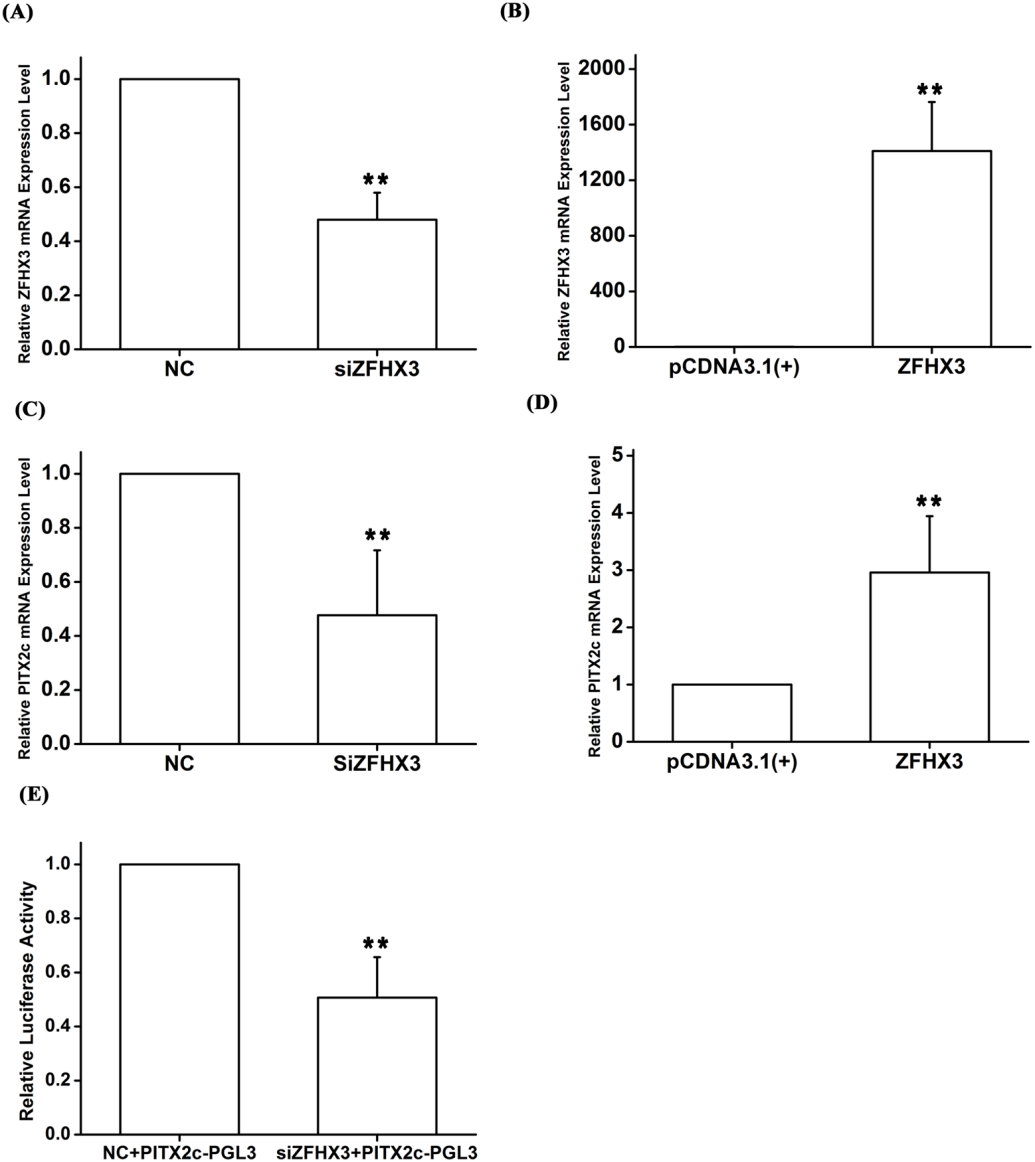
The *ZFHX3* gene positively regulates expression of the *PITX2c* gene. HCT116 cells were transfected with siRNA specific for *ZFHX3* or an expression plasmid for *ZFHX3* and used for isolation of total RNA samples, real time RT-PCR analysis and Luciferase assays. A. Real-time RT-PCR analysis for *ZFHX3*. Transfection of siRNA for *ZFHX3* successfully reduced expression of *ZFHX3*. B. Real-time RT-PCR analysis for *ZFHX3*. Transfection of an expression plasmid for *ZFHX3* successfully increased expression of *ZFHX3*. C. Real-time RT-PCR analysis for *PITX2c*. Transfection of siRNA for *ZFHX3* reduced expression of *PITX2c*. D. Real-time RT-PCR analysis for *PITX2c*. Transfection of an expression plasmid for *ZFHX3* successfully increased expression of *PITX2c*. E. Luciferase assays for the *PITX2c* promoter activity in cells transfected with a siRNA specific for *ZFHX3* or a control scramble siRNA. *P<0.05; **P<0.01.

The AF SNP rs2106261 identified by GWAS is located within the *ZFHX3* gene, therefore, we consider *ZFHX3* as a strong candidate gene for AF at the chromosome 16q22 locus. As our genetic studies indicate a gene-gene interaction between *PITX2c* and *ZFHX3*, we hypothesized that *ZFHX3* may regulate expression of *PITX2c*. Interestingly, knockdown of *ZFHX3* expression by a specific siRNA significantly decreased expression of *PITX2c* about 2-fold (*P* = 5.00×10^-3^) (Fig 4A and 4C). Conversely, overexpression of *ZFHX3* significantly increased expression of *PITX2c* by 2.96-fold (*P* = 2.00×10^-3^) (Fig 4B and 4D). Knockdown of *ZFHX3* expression by siRNA reduced the transactivation activity from a reporter with a 1.5 kb DNA fragment upstream of the *PITX2c* transcriptional start site fused to the luciferase gene (PITX2c-PGL3) by 1.97-fold (*P* = 5.00×10^-3^) (Fig 4E).

### Molecular basis of gene-gene interaction: Both *PITX2c* and *ZFHX3* positively regulate the expression of *NPPA*

**Fig 5:**
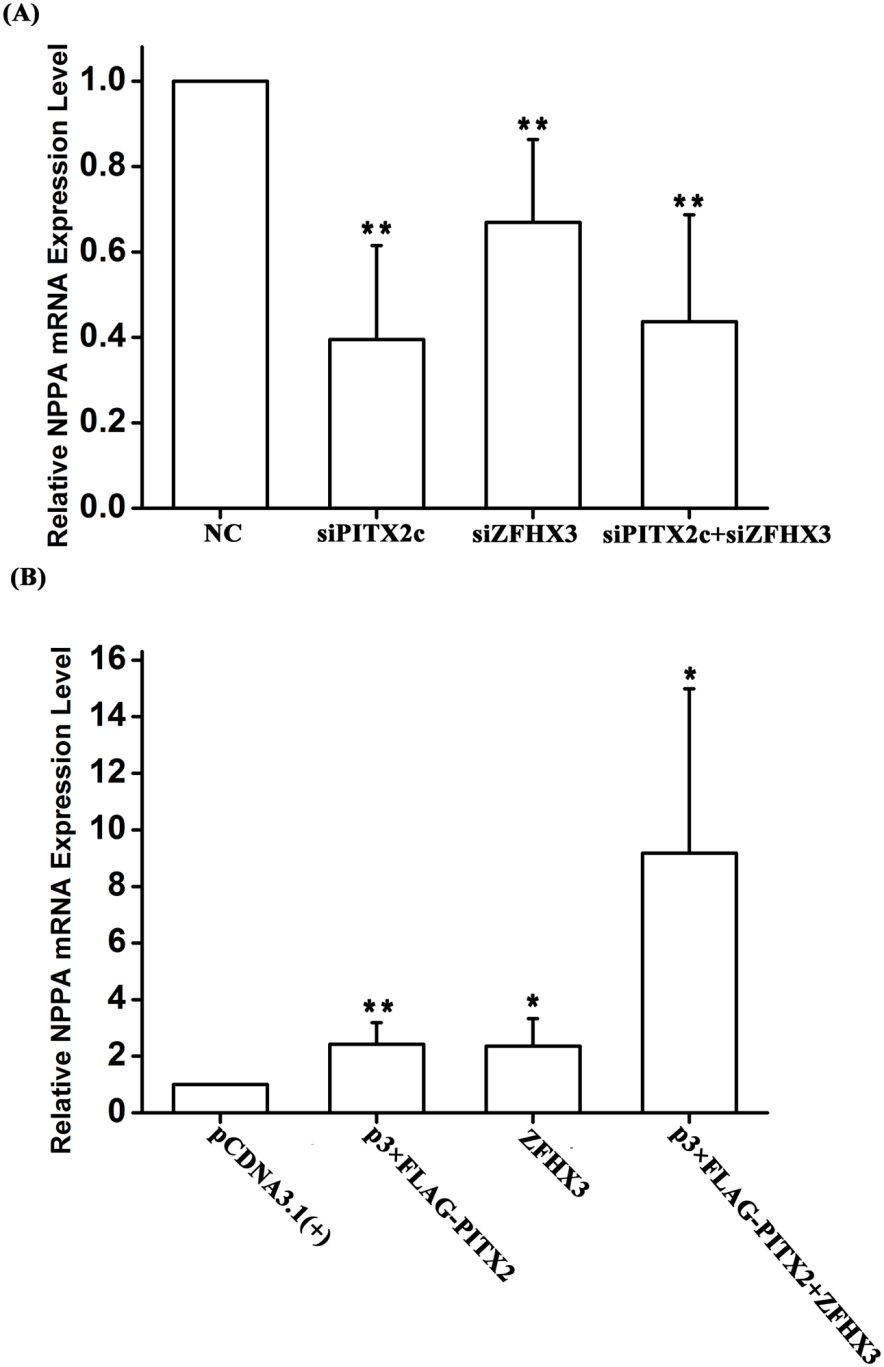
Both *PITX2c* and *ZFHX3* positively regulate expression of the *NPPA* transcription. HCT116 cells were co-transfected with an expression plasmid for either *PITX2c*, *ZFHX3* or both, or siRNA for either *PITX2c*, *ZFHX3* or both and used for measurements of RT-PCR. A. Knockdown of *PITX2c*, *ZFHX3* or both by siRNAs down-regulated *NPPA* expression. B. Overexpression of either *PITX2c* or *ZFHX3* up-regulated *NPPA* expression. Co-expression of both *PITX2c* and *ZFHX3* dramatically increased *NPPA* expression. *P<0.05; **P<0.01.

Several earlier studies showed that *PITX2c* regulates the expression of the *NPPA* gene encoding ANF (a cardiac protein hormone), but conflicting results on either positive regulation or negative regulation were obtained in different studies [13,15,16]. We tested the regulation of *NPPA* by *PITX2c* in HCT116 cells. As showed in Fig 5A, knockdown of *PITX2c* expression using siRNA significantly reduced expression of *NPPA* by 60% (*P* = 3.20×10^-4^). Overexpression of *PITX2c* by transfection of HCT116 cells with p3×FLAG-PITX2c significantly increased *NPPA* expression by 2.42 fold (*P* = 0.01) (Fig 5B).

Interestingly, we found that *ZFHX3* also regulated *NPPA* expression. As shown in Fig 5A, knockdown of *ZFHX3* expression by siRNA significantly decreased expression of *NPPA* (*P* = 4.00×10^-3^). Overexpression of *ZFHX3* up-regulated *NPPA* expression 2.36 fold (*P* = 0.03) (Fig 5B).

Overexpression of both *PITX2c* and *ZFHX3* dramatically increased expression of *NPPA* (*P* = 0.031) (Fig 5B). Knockdown of both *PITX2c* and *ZFHX3* also reduced expression of *NPPA* (*P* = 1.00×10^-3^) (Fig 5A).

**Fig 6:**
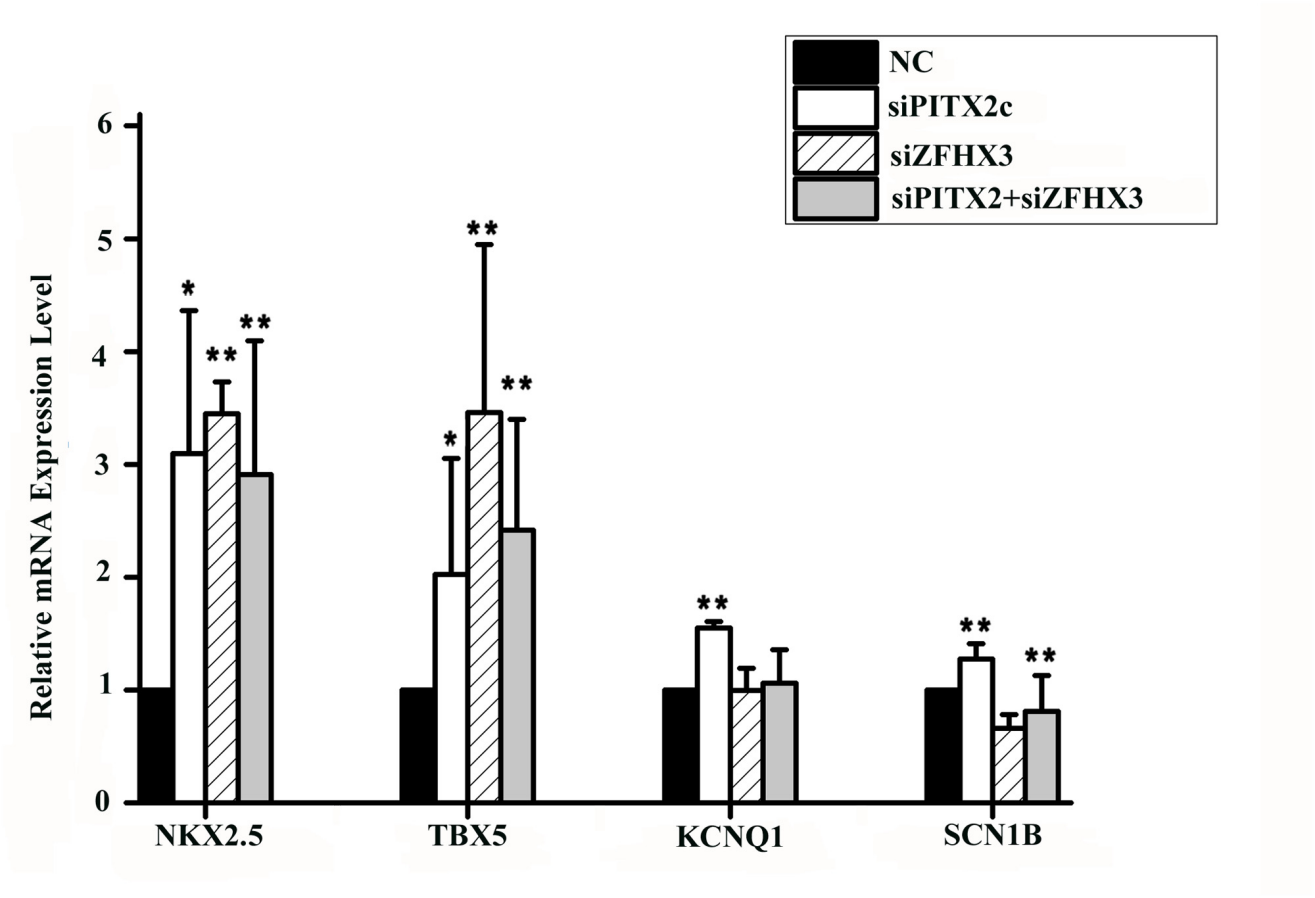
*PITX2c* and *ZFHX3* regulate expression of *NKX2*.*5*, *TBX5*, *KCNQ1* and *SCN1B*. HCT116 cells were transfected with siRNA specific for *PITX2c* or *ZFHX3* and used for isolation of total RNA samples and real-time RT-PCR analysis. Transfection of siRNA for *PITX2c* increased expression of *NKX2*.*5*, *TBX5*, *KCNQ1* and *SCN1B*. Transfection of siRNA for *ZFHX3* increased expression of *NKX2*.*5* and *TBX5*,but decreased expression of *SCN1B*. *ZFHX3* did not affect on the expression of *KCNQ1*. Transfection of siRNAs for both *PITX2c* and *ZFHX3* increased expression of *NKX2*.*5* and *TBX5*.

It was reported that *PITX2c* could also regulate expression of other downstream genes including *NKX2*.*5* (encoding NK2 transcription factor related, locus 5), *TBX5* (encoding T-box 5), *KCNQ1* (encoding potassium voltage-gated channel, KQT-like subfamily, member 1), and *SCN1B* (encoding sodium channel, voltage-gated, type I, beta subunit) [13,15,17,18]. As shown in Fig 6, knockdown of the *PITX2c* expression by siRNA significantly increased expression of *NKX2*.*5* by 3.10-fold, *TBX5* by 2.32-fold, *KCNQ1* by 1.55-fold, and *SCN1B* by 1.27-fold. Interestingly, knockdown of the *ZFHX3* gene by siRNA also significantly increased expression of *NKX2*.*5* by 3.45-fold and *TBX5* by 3.23-fold, but decreased expression of *SCN1B* by 1.52-fold and did not affect expression of *KCNQ1* (Fig 6). Co-transfection of both *PITX2c* siRNA and *ZFHX3* siRNA also significantly reduced *NKX2*.*5* by 2.91-fold, *TBX5* by 2.42-fold, but did not affect expression of *KCNQ1* or *SCN1B* (Fig 6).

### No significant gene-gene interaction between *ZFHX3* variant rs2106261 and *CAV1* variant rs3807989 or between *PITX2c* variant rs2200733 *CAV1* variant rs3807989

GWAS in European ancestry populations have identified ten genetic loci for AF [5,7,8,10,11]. We analyzed these loci in the Chinese Han population for their association with AF. We found that in addition to the *ZFHX3* locus and the *PITX2c* locus reported previously [6,9], one other locus, rs3807989 in *CAV1* encoding caveolin-1, also showed significant association with AF, whereas no significant association was identified for other loci [12]. Therefore, we also analyzed gene-gene interactions between rs2106261 and rs3807989 and between rs2200733 and rs3807989. The classical gene-gene analysis by comparing ORs for the nine two-locus genotypes did not reveal any significant synergistic effect between rs2106261 and rs3807989 (Table 2). The OR for the double risk homozygotes for both rs2106261 and rs3807989 (AAGG) was 1.25, which is smaller than the product of the ORs (1.18+1.15) for each single-risk homozygotes (AAAA, homozygous risk genotype for rs2106261; GGGG, homozygous risk genotype for rs3807989) (Table 2). These results suggest that there is no interaction between the *ZFHX3* locus and the *CAV1* locus for AF. Similarly, the OR for the double risk homozygotes for both rs2200733 and rs3807989 (TTGG) was 1.08, which is smaller than the product of the ORs (1.00+0.77) for each single-risk homozygotes (TTAA, homozygous risk genotype for rs2200733; CCGG, homozygous risk genotype for rs3807989) (Table 2). These results suggest that there is no interaction between the *PITX2c* locus and the *CAV1* locus for AF.

**Table 2:** ORs for 8 two-locus genotypes versus non-risk homozygous genotype AAGG as a reference for SNPs rs2106261 and rs3807989, CCAA as a reference for SNPs rs2200733 and rs3807989 in the Chinese Han population.

Two-locus genotype				Allelic test^a^		Adjust for age and gender^b^	
rs2106261	rs3807989	N of cases (%)	N of controls (%)	P value	OR (95%CI)	P value	OR (95%CI)
1,578 cases /2,389 controls							
GG	AA	44 (1.1%)	110 (4.6%)	N.A‡	1.00	N.A	1.00
GG	AG	221 (5.6%)	442 (18.5%)	0.255	1.25 (0.85–1.84)	0.79	1.06 (0.69–1.63)
GG	GG	357 (22.6%)	577 (24.2%)	0.021	1.55 (1.06–2.25)	0.19	1.15 (0.93–1.42)
AG	AA	47 (3.0%)	93 (3.9%)	0.354	1.26 (0.77–2.78)	0.66	1.04 (0.87–1.25)
AG	AG	253 (16.0%)	383 (16.0%)	0.10	1.64 (1.12–2.44)	0.02	1.27 (1.03–1.54)
AG	GG	403 (25.5%)	520 (21.8%)	4.34×10^-4^	1.92 (1.33–2.78)	4.58×10^-4^	2.04 (1.37–3.03)
AA	AA	18 (1.1%)	14 (0.6%)	3.00×10^-3^	3.23 (1.47–7.14)	0.02	1.18 (1.03–1.35)
AA	AG	78 (4.9%)	97 (4.1%)	0.003	2.01 (1.27–3.23)	8.00×10^-3^	1.15 (1.03–1.27)
AA	GG	157 (9.9%)	153 (6.4%)	6.23×10^-6^	2.57 (1.70–3.88)	6.62×10^-5^	1.25 (1.12–1.40)
1,578 cases /2,389 controls							
**rs2200733 rs3807989**							
CC	AA	24 (1.5%)	43 (1.8%)	N.A	1.00	N.A	1.00
CC	AG	79 (5.0%)	234 (9.8%)	0.08	0.61 (0.345–1.06)	0.03	0.50 (0.262–0.94)
CC	GG	121 (7.7%)	317 (13.3%)	0.17	0.68 (0.40–1.18)	0.09	0.77 (0.568–1.04)
CT	AA	44 (6.9%)	111 (4.6%)	0.27	0.71 (0.386–1.31)	0.15	0.85 (0.68–1.06)
CT	AG	271 (17.2%)	485 (20.3%)	1.00	1.00 (0.60–1.69)	0.53	0.95 (0.83–1.10)
CT	GG	454 (28.8%)	650 (27.2%)	0.39	1.25 (0.75–2.09)	0.82	0.99 (0.88–1.11)
TT	AA	41 (2.6%)	63 (2.6%)	0.64	1.17 (0.62–2.20)	0.96	1.00 (0.88–1.12)
TT	AG	202 (12.8%)	203 (8.5%)	0.03	1.78 (1.04–3.05)	0.30	1.05 (0.96–1.41)
TT	GG	342 (21.7%)	283 (11.8%)	3.00×10^-3^	2.17 (1.28–3.66)	0.03	1.08 (1.01–1.16)

OR: odds ratio; CI: confidence interval

^a^nominal *P* value and OR computed using Chi-square tests with Pearson’s 2×2 contingency tables.

^b^nominal *P* value and OR computed using multivariable logistic regression analysis including age and gender as covariates.

**Fig 7:**
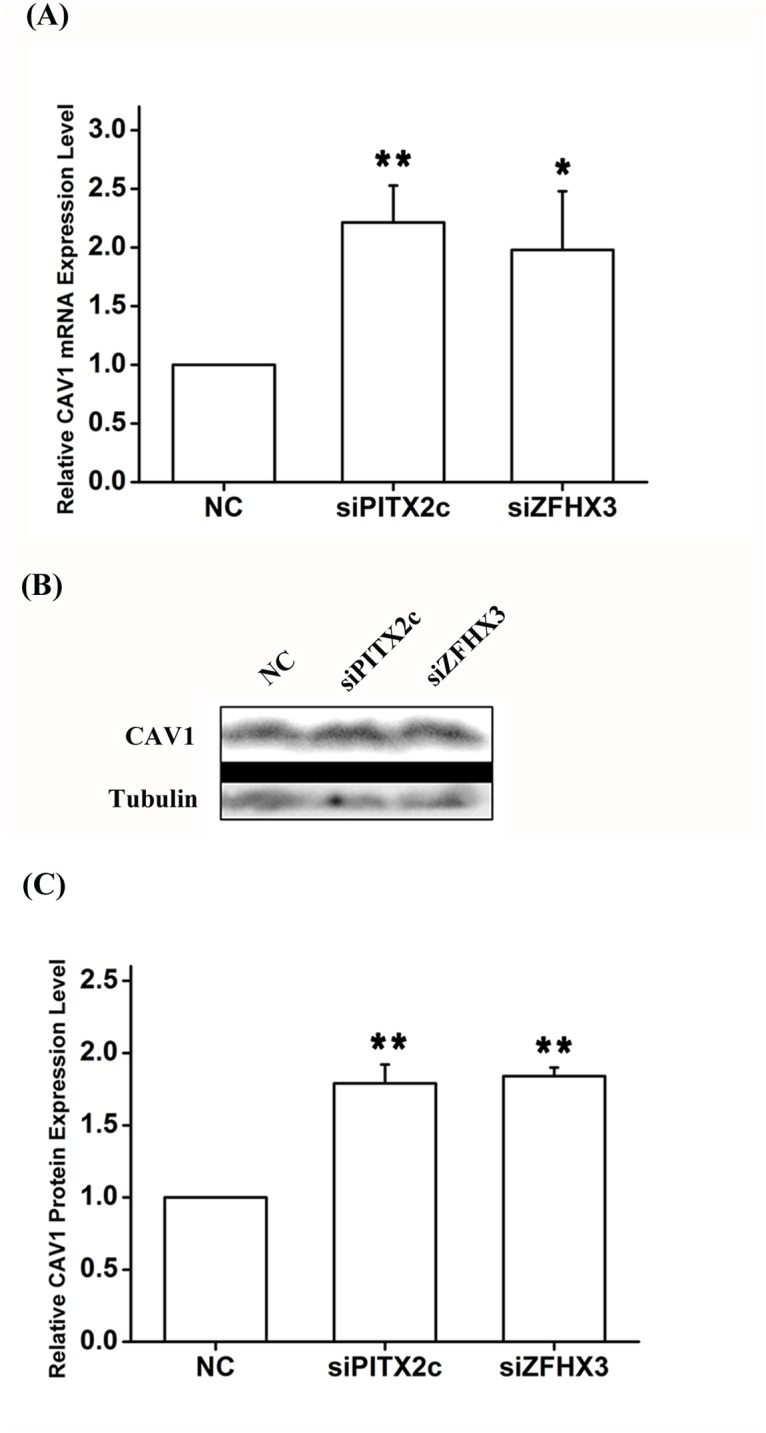
*PITX2c* and *ZFHX3* negatively regulate expression of the *CAV1* gene. HCT116 cells were transfected with siRNA specific for *PITX2c* or siRNA specific for *ZFHX3* and used for isolation of total RNA samples and real-time RT-PCR analysis. A. Real-time RT-PCR analysis for *CAV1*. Transfection of siRNA for *PITX2c* or siRNA for *ZFHX3* successfully increased expression of *CAV1*. B, C. Western blot analysis revealed that *PITX2c* and *ZFHX3* increased the expression of the CAV1 protein by 1.79-fold and 1.84-fold, respectively (*P* = 2.21×10^-5^, 2.00×10^-7^). **P<0.01; *<0.05.

**Fig 8:**
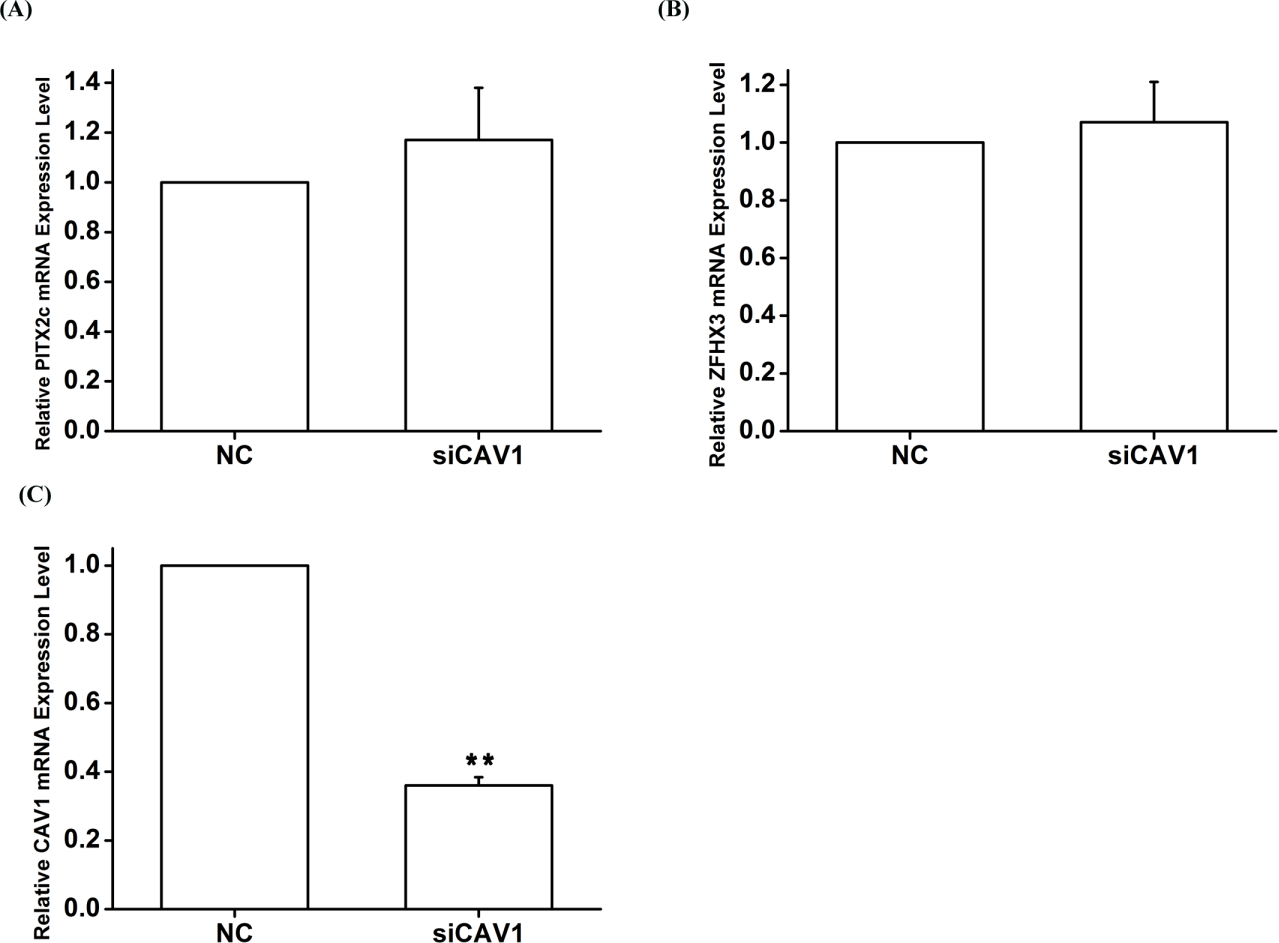
The *CAV1* gene does not affect expression of *PITX2c* or *ZFHX3*. HCT116 cells were transfected with siRNA specific for *CAV1* and used for isolation of total RNA samples and real-time RT-PCR analysis. A. Real-time RT-PCR analysis for *PITX2*. Transfection of siRNA for *CAV1* did not significantly affect the expression of *PITX2*. B. Real-time RT-PCR analysis for *ZFHX3*. Transfection of siRNA for *CAV1* did not significantly affect the expression of *ZFHX3*. *C*. Real-time RT-PCR analysis for *CAV1*. Transfection of siRNA for *CAV1* successfully reduced expression of *CAV1*.**P<0.01; *<0.05.

Real-time RT-PCR analysis showed that knockdown of either *ZFHX3* or *PITX2c* increased the expression level of *CAV1* (Fig 7). Similar results were obtained with Western blot analysis (Fig 7). On the contrary, knockdown of *CAV3* did not significantly affect the expression of *ZFHX3* or *PITX2c* (Fig 8). Together, these data suggest that there is no cyclic cross-regulation between *ZFHX3* and *CAV1* or between *PITX2c* and *CAV1*.

### Analysis of gene-gene interactions by alternative gene-gene interaction programs

Many gene-gene programs have been developed in recent years, therefore, we also analyzed interaction among SNPs rs2200733/*PITX2c*, rs2106261/*ZFHX3* and rs3807989/*CAV1* using RERI and INTERSNP programs. RERI (relative excess risk due to interaction) analysis was developed to quantify the extent of synergistic effect by adopting a fundamental measure of additive interaction and relative excess risk due to interaction (RERI) [19]. Here we used this strategy to investigate interaction between rs2106261 and rs2200733 in terms of risk alleles A and T in the combined population. The RERI analysis can distinguish the additive effect from the synergistic effect [19]. A significant RERI value higher or lower than 0 is considered to demonstrate a synergistic effect, whereas a non-significant RERI value indicates an additive effect [19]. The results are shown in Table 3. First, we analyzed the synergistic effect when exposed to one copy of risk alleles at any one locus or both loci (H1). No significant synergistic effect was observed between rs2106261 and rs2200733 (RERI = 0.22 (95% CI -0.20–0.54), *P* = 0.13; RERI = 0.18 (95% CI -0.29–0.52), *Padj* = 0.22 after adjustment of covariates of age and gender). Second, we assessed the synergistic effect when exposed to two copies of risk alleles at any one locus or both loci (H2). A significant synergistic effect was detected between rs2106261 and rs2200733 with a RERI value of 2.26 (95% CI 1.06–3.73) (*P<1*.*00*×10^-4^; RERI = 2.87 (1.48–4.69), *Padj<1*.*00*×10^-4^ after adjustment of covariates of age and gender). Third, we assessed the synergistic effect when exposed to two copies of risk alleles at one locus and one copy of risk alleles at the other locus (H3). A significant synergistic effect was detected between rs2106261 and rs2200733 with a RERI value of 0.99 (95% CI 0.29–1.79) (*P<1*.*00*×10^-4^; RERI = 1.29 (95% CI 0.44–2.33), *Padj<1*.*00*×10^-4^ after adjustment of covariates of age and gender). These results provided statistical genetic evidence for the interaction between rs2106261 and rs2200733.

**Table 3:** Interaction between SNPs rs2106261 and rs2200733 in the Chinese Han population.

Exposed	Genotype		N of case	N of control	RERI (95%CI)	*P* ^b^	RERI_adj_ (95%CI) ^a^	*P* ^b^
	rs2106261	rs2200733						
	2,020 cases /5,315 controls							
H1	AG	CT	445	1202	0.22 (-0.20–0.54)	0.13	0.18(-0.29–0.52)	0.22
	GG	CT	396	1267				
	AG	CC	121	534				
	GG	CC	131	605				
H2	AA	TT	148	141	2.26 (1.06–3.73)	<1.00×10^-4^	2.87(1.48–4.69)	<1.00×10^-4^
	GG	TT	278	579				
	AA	CC	38	128				
	GG	CC	131	605				
H3	AA	TT	148	141	0.99 (0.29–1.79)	<1.00×10^-4^	1.29(0.44–2.33)	<1.00×10^-4^
	AG	TT	319	566				
	AA	CT	144	293				
	AG	CT	445	1202				

H1: Exposed to one copy of risk alleles; H2: Exposed to two copies of risk allele; H3: Exposed to one additional copy of risk allele

^a^RERI_adj_ were computed by adjusting for age and gender.

^b^ *P* values were estimated by 10000 times of bootstrap sampling.

The RERI analysis did not identify any significant interaction between *ZFHX3* SNP rs2106261 and *CAV1* variant rs3807989 (H1: RERI = 0.51 (95% CI -0.54–1.12), *Padj* = 0.19; H2: RERI = -0.52 (95% CI -5.64–1.41), *Padj* = 0.37; H3: RERI = 0.29 (95% CI -0.61–1.06), *Padj* = 0.36) (Table 4). The RERI analysis was also used to analyze the interaction between *PITX2c* variant rs2200733 and *CAV1* variant rs3807989 (H1: RERI = 0.80 (95% CI -0.02–1.27), *Padj* = 0.11; H2: RERI = 1.37 (95% CI 0.24–2.71), *Padj* = 0.05; H3: RERI = 0.42 (95% CI -0.33–1.12), *Padj* = 0.08).

**Table 4:** Interaction analysis of rs2106261 vs. rs3807898 and rs2200733 vs. rs3807898 in the Chinese Han population.

Exposed	Genotype		N of case	N of control	RERI (95%CI)	*P* ^b^	RERI_adj_ (95%CI) ^a^	*P* ^b^
	**rs2106261**	**rs3807989**						
	1,578 cases /2,389 controls							
H1	AG	AG	253	383	0.14 (-1.07–0.75)	0.71	0.51 (-0.54–1.12)	0.19
	GG	AG	221	442				
	AG	AA	47	93				
	GG	AA	44	110				
H2	AA	GG	157	153	-1.20 (-6.36–0.80)	0.09	-0.52 (-5.64–1.41)	0.37
	GG	GG	357	577				
	AA	AA	18	14				
	GG	AA	44	110				
H3	AA	GG	157	153	0.16 (-0.59–0.81)	0.60	0.29(-0.61–1.06)	0.36
	AG	GG	403	520				
	AA	AG	78	97				
	AG	AG	253	383				
	**rs2200733**	**rs3807989**						
	1,578 cases /2,389 controls							
H1	CT	AG	271	485	0.69 (-0.22–1.14)	0.15	0.80(-0.02–1.27)	0.11
	CC	AG	79	234				
	CT	AA	44	111				
	CC	AA	24	43				
H2	TT	GG	342	283	1.32 (0.15–2.58)	0.04	1.37(0.24–2.71)	0.05
	CC	GG	121	317				
	TT	AA	41	63				
	CC	AA	24	43				
H3	TT	GG	342	283	0.13 (-0.58–0.76)	0.56	0.42 (-0.33–1.12)	0.08
	CT	GG	319	566				
	TT	AG	454	650				
	CT	AG	271	485				

H1: Exposed to one copy of risk alleles; H2: Exposed to two copies of risk allele; H3: Exposed to one additional copy of risk allele

^a^RERI_adj_ were computed by adjusting for age and gender.

^b^ *P* values were estimated by 10000 times of bootstrap sampling.

We also analyzed gene-gene interaction using the INTERSNP program[20,21], which can analyze genotypic interactions under additive by additive, additive by dominant, dominant by additive and dominant by dominant terms. For rs2106261 and rs2200733, nominal significant interaction was found additive × additive after adjusting for age and gender (OR = 0.85, 95% CI: 0.74–0.97, *Padj* = 0.02), although the global test on all interaction terms were not significant (Table 5). After simplifying the model by removing dominant effects without significant loss of goodness-of-fit of the model (*P* = 0.11), the additive interaction on a multiplicative OR scale was also significant (OR = 0.85, 95% CI: 0.76–0.96, *Padj* = 0.01) (Table 5). A similar pattern was found for additive × additive interaction between rs2200733 and rs3807989 under models with dominant effects (OR = 1.40, 95% CI: 1.16–1.70, *Padj* = 1.00×10^-3^) and after removing dominant effects (OR = 1.25, 95% CI: 1.06–1.48, *Padj* = 7.00×10^-3^). No significant genotypic interaction was found for rs2106261 and rs3807989 under any model (Table 5).

**Table 5:** Genotypic interaction with logistic regression by INTERSNP developed by Cordell and Clayton [20,21].

**SNP Pair: rs2106261 by rs2200733**				
**Genotypic interaction (test #6)**	**OR (95%CI)** ^**a**^	***P*** ^**a**^	**OR**_**adj**_ **(95%CI)** ^**b**^	***P***_***adj***_ ^**b**^
A×A	0.89 (0.79–1.01)	0.06	0.85 (0.74–0.97)	0.02
A×D	0.95 (0.81–1.13)	0.57	0.95 (0.79–1.14)	0.55
D×A	1.06 (0.89–1.26)	0.50	1.09 (0.90–1.31)	0.39
D×D	1.12 (0.89–1.42)	0.34	1.07 (0.83–1.38)	0.60
		Global *P* = 0.23		Global *P* = 0.11
**Additive interaction model (test #5)**				
A×A	0.89 (0.80–1.00)	0.04	0.85 (0.76–0.96)	0.01
**SNP Pair: rs2106261 by rs3807989**				
**Genotypic interaction (test #6)**	**OR (95%CI)**	***P***	**OR**_**adj**_ **(95%CI)**	***P***_***adj***_
A×A	1.18 (0.96–1.45)	0.12	1.09(0.87–1.37)	0.47
A×D	0.84(0.64–1.09)	0.18	0.88(0.65–1.19)	0.41
D×A	0.85(0.65–1.13)	0.25	0.78(0.57–1.06)	0.11
D×D	1.26(0.88–1.81)	0.22	1.46(0.97–2.19)	0.07
		Global *P* = 0.52		Global *P* = 0.44
**Additive interaction model (test #5)**				
A×A	1.06 (0.92–1.23)	0.43	0.99 (0.84–1.17)	0.95
**SNP Pair: rs2200733 by rs3807989**				
**Genotypic interaction (test #6)**	**OR (95%CI)**	***P***	**OR**_**adj**_ **(95%CI)**	***P***_***adj***_
A×A	1.28(1.08–1.52)	4.41×10^-3^	1.40(1.16–1.70)	1.00×10^-3^
A×D	0.81(0.64–1.02)	0.07	0.79(0.61–1.03)	0.08
D×A	0.80(0.62–1.03)	0.08	0.76(0.57–1.00)	0.05
D×D	1.17(0.84–1.64)	0.35	1.37(0.95–1.99)	0.09
		Global *P* = 0.05		Global *P* = 9.00×10^-3^
**Additive interaction model (test #5)**				
A×A	1.16 (1.00–1.34)	0.06	1.25 (1.06–1.48)	7.00×10^-3^

A: Additive; D: Dominant; OR: odds ratio; 95% CI: 95% confidence interval.

^a^OR and 95% CI before adjustment for age and gender.

^b^OR and 95% CI after adjustment for age and gender.

## Discussion

In this study, we show that gene-gene interaction plays an important role in generation of disease phenotype by identifying gene-gene interaction involved in the pathogenesis of a cardiac disorder, AF. We employed a multi-stage case control association design to compare the frequencies of all nine two-locus genotypes from GWAS SNPs rs2106261 in the *ZFHX3* gene and rs2200733 close to the *PITX2c* gene. Our study involves a careful design with a Discovery population consisting of 569 cases and 1,996 controls, Replication I population with 641 cases and 1,692 controls, and Replication II population composed of 810 cases and 1,627 controls. The combined population has 2,020 cases and 5,315 controls, and is considered to represent a considerably large sample size in the modern population studies for AF. We consider this point as strength of this study. When SNP rs2106261 and rs2200733 were analyzed together, two-locus genotype AATT (double risk homozygote) showed the highest odds ratio (OR) of 6.64 (95% CI 3.64–12.11) (*P* = 6.38×10^-10^), 4.04 (95% CI 2.23–7.32) (*P* = 4.34×10^-6^), 5.70 (95% CI 3.34–9.71) (*P* = 1.58×10^-10^) and 5.36 (95% CI 3.87–7.43) (*P* = 8.00×10^-24^) in the Discovery, Replication I, II, and combined population, respectively, when compared to wild type non-risk genotype GGCC. The Breslow-Day test showed that the ORs for AATT were significantly higher than ORs for GGTT or AACC in all populations (*P* = 5.26×10^-5^ vs. GGTT and 2.94×10^-22^ vs. AACC in the combined population) and higher than the combined ORs for both GGTT and AACC (5.36 vs. 3.31 in the combined population). We also analyzed gene-gene interaction using the RERI analysis and identified synergistic effects between SNP rs2106261 and rs2200733 when exposed two copies of risk alleles at any one locus or both loci (H2) (*P<1*.*00*×10^-4^) or when exposed to two copies of risk alleles at one locus and one copy of risk alleles at the other locus (H3) (*P<1*.*00*×10^-4^) (Table 3). Analysis using the INTERSNP program revealed significant genotypic interaction between SNP rs2106261 and rs2200733 under an additive × additive model, but not under other models (Table 5). Overall, our studies establish that gene-gene interaction is involved in the pathogenesis of AF. Most importantly, our results suggest that gene-gene interaction accounts for heritability of human disease because it generates synergistic effects that markedly increase disease risk.

The present study identifies the interaction between two common GWAS loci for AF. Ritchie et al [22] previously found that the risk alleles of common variants rs2200733 and rs10033464 at the 4q25 *PITX2c* AF locus could predict whether carriers of rare mutations in *SCN5A* (encoding the cardiac sodium channel), *NPPA*, *KCNA5* (encoding potassium voltage-gated channel, shaker-related subfamily, member 5), and *NKX2*.*5* (encoding transcriptional factor NK2 homeobox 5) developed AF, suggesting potential interaction between common variants and rare mutation in familial AF. Moreover, Lubitz et al [23] studied AF risk signals within nine GWAS loci and found that there are at least four distinct AF susceptibility signals at the 4q25 AF locus upstream of *PITX2c* that may increase the risk of AF by 5-fold together.

**Fig 9:**
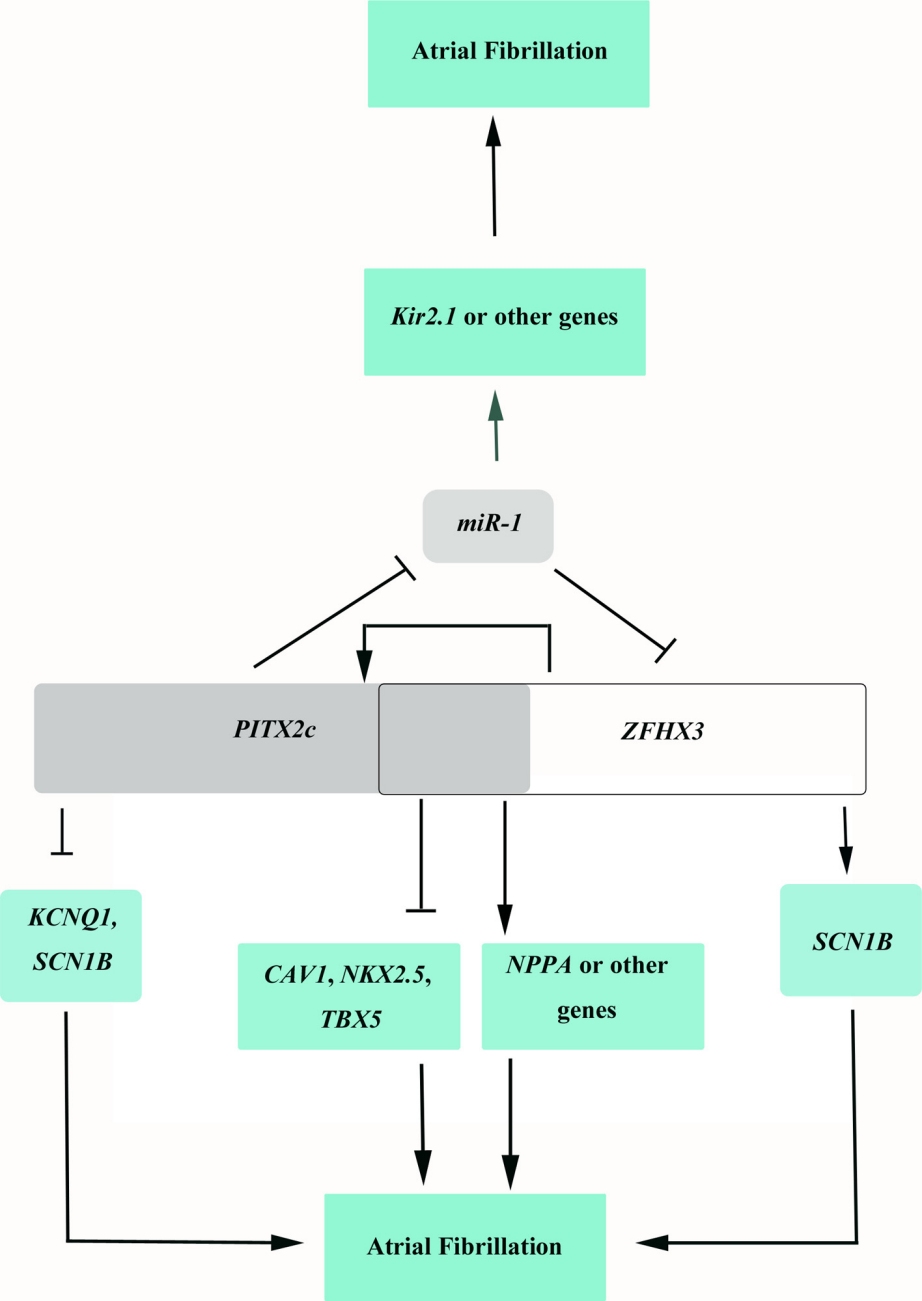
A schematic diagram showing the cyclic cross-regulation of *PITX2c* and *ZFHX3*, which underlies the interaction between *PITX2c* and *ZFHX3* involved in the pathogenesis of AF.

Our cellular and molecular biological studies here on rs2200733/*PITX2c* and rs2106261/*ZFHX3* identify a fundamental molecular mechanism underlying gene-gene interaction. SNP rs2200733 on 4q25 was the first genomic variant for AF identified by GWAS [5] and located 146 kb from the *PITX2c* gene encoding a paired-like homeodomain transcription factor 2 involved in the asymmetrical development of the heart and other organs [24–26]. Heterozygous knockout *PITX2c* mice developed atrial arrhythmias (atrial flutter, atrial tachycardia) upon programmed stimulation [13]. Kirchhof et al [14] showed that *PITX2c* is expressed in human and mouse left atria. Isolated hearts from heterozygous *PITX2c* knockout mice developed AF upon programmed stimulation and showed shortened action potential duration [14]. Chinchilla et al [15] showed that the expression level of *PITX2c* was decreased in AF patients and that atria-specific, but not ventricle-specific knockout of *PITX2c*, resulted in differences in action potential amplitude and increased expression of *miR-1*. Therefore, all evidence to date strongly suggests that *PITX2c* should be the causative gene for AF at the 4q25 locus. SNP rs2106261 is located within the *ZFHX3* gene. *ZFHX3* encodes a transcription factor [27] which contains four homeodomains and seventeen zinc fingers [28]. The ZFHX3 transcription factor appears to regulate myogenic [29] and neuronal differentiation [30]. Although the function of *ZFHX3* in cardiac tissue is unknown, it is expressed in mouse hearts [31]. Here we show that *PITX2c* and *ZFHX3* positively cross-regulates each other. *PITX2c* negatively regulates expression of *miR-1*, which negatively regulates expression of *ZFHX3* by targeting a *miR-1*-binding site at the 3’-UTR, resulting in a positive regulation of *ZFHX3* by *PITX2c* (Fig 9). Interestingly, *ZFHX3* positively regulates expression of *PITX2c*. The net effect is a cyclic loop of cross-regulation between *ZFHX3* and *PITX2c* (Fig 9). A cyclic loop of cross-regulation of two risk genes for a disease is expected to generate synergistic effects, which further increase disease risk, and therefore provides a novel molecular mechanism for gene-gene interaction. One important future direction is to determine whether this novel mechanism applies to other human disease and to plant and animal phenotypes in general.

On the molecular level, how does the cyclic loop of cross-regulation between *ZFHX3* and *PITX2c* generate interaction between the two genes and increase risk of AF? The expression level of *miR-1* was shown to be reduced in human AF patients, which was correlated with up-regulation of potassium channel Kir2.1 and increased potassium current I_K1_ responsible for AF maintenance. *PITX2c* negatively regulates expression of *miR-1*, which increases I_K1_, resulting in AF. Decreased *miR-1* increases expression of *ZFHX3*, which increases expression of *PITX2c*, further decreases expression of *miR-1* and increases risk of AF (Fig 9). *ZFHX3* increases expression of *PITX2c*, which decreases expression of *miR-1* and increases risk of AF (Fig 9). *PITX2c* positively regulates expression of *ZFHX3*, which further increases expression of *PITX2c*, and leads to down-regulation of *miR-1* expression and increased risk of AF (Fig 9). In addition to *miR-1*, *PITX2c* and *ZFHX3* may regulate *NPPA*, *TBX5*, *NKX2*.*5* or other downstream target genes to increase risk of AF (Fig 9). These results provide novel insights into the roles of gene-gene interaction in the pathogenesis of AF.

One other important insight from this study is that not all risk genes for AF interact each other. We have previously shown that genomic variants increase susceptibility of cardiovascular diseases in a population-specific manner. Although some variants increase disease risk in both European ancestry populations and Asian populations, but other variants show significant disease association only in Asian populations [9,11,32]. For AF, we found that among ten GWAS variants for AF identified in European ancestry populations, only three were associated with risk of AF in the Chinese population, including SNPs rs2106261 in the *ZFHX3* gene, rs2200733 at the *PITX2c* locus, and rs3807989 in the *CAV1* gene. Despite the robust gene-gene interaction identified for rs2106261/*ZFHX3* gene and rs2200733/*PITX2c*, we did not identify any significant interaction between rs2106261/*ZFHX3* and rs3807989/*CAV1* with all three gene-gene interaction programs (Tables 2, 4 and 5). For interaction between rs2200733/*PITX2c* and rs3807989/*CAV1*, inconsistent results were obtained. Analysis for the OR for each multi-locus genotype and RERI analysis did not find gene-gene interaction between rs2200733 and rs3807989, whereas the INTERSNP program found significant interaction under a model of additive by additive. Future studies are needed to reconcile the differences between different programs developed for studying gene-gene interaction.

One limitation of the present study is that our statistical analysis was not adjusted for principal components to correct for possible stratification in Chinese samples due to a limited number of SNPs genotyped in the study populations. Genetic interaction may be especially susceptible to small degrees of population stratification, however, this may be unlikely given the replication of the finding in multiple populations.

In summary, we have found that gene-gene interaction can generate synergistic effects that markedly increase disease risk, therefore, accounting for a portion of heritability of human disease. Our identification of the gene-gene interaction between SNPs rs2106261 in the *ZFHX3* gene and rs2200733 at the *PITX2c* locus provide significant insights into the pathogenesis of AF. We further show that *PITX2c* and *ZFHX3* positively regulate each other at the molecular level, generating a loop of cross-regulation between *PITX2c* and *ZFHX3*. Our data provide an interesting molecular basis for some gene-gene interaction at the molecular genetic level.

## Materials and Methods

### Study subjects and preparation of genomic DNA samples

The subjects involved in the present study include AF patients and non-AF controls selected from the GeneID database [6,9,32–39]. All study subjects are of Han ethnic origin based on self-description. The study was approved by the Ethics Committee of Huazhong University of Science and Technology and the Ethics Committees from local hospitals, and consistent with the guideline in the Declaration of Helsinki. Written informed consent was obtained from the participants.

The diagnosis of AF was made by multiple experienced cardiologists and cardiac electrophysiologists using data from 12-lead surface electrocardiograms (ECGs) or Holter recordings. The ECG characteristics of AF include the absence of P waves, the presence of rapid oscillations or fibrillatory waves (F waves), and irregular R-R intervals [40–42]. The controls are healthy individuals who do not have AF at the time of physical examinations or from medical records.

Details of study subjects and GeneID and preparation of genomic DNA samples were described in S1 Text.

### Genotyping of SNPs

Genotyping of SNPs was carried out using High-Resolution Melt (HRM) analysis as described previously by us [6,9,32–39]. HRM genotyping data were validated by direct sequencing analysis of 52 randomly selected study subjects. Primers for genotyping are listed in S6 Table. The HRM genotyping data matched the sequencing data.

### Prediction of potential miR-1 binding sites

Details of bioinformatics prediction of miR-1 binding sites were described in S1 Text.

### Plasmids, siRNAs, and microRNA mimics

Details of plasmids, siRNAs and microRNA mimics were described in S1 Text.

### Cell culture and dual luciferase reporter assays

HCT116 and SW620 cells were cultured and transfected with plasmid DNA, siRNAs, and microRNA mimics using Lipofectamine 2000 and the Opti-MEM I reduced serum medium as described [43,44]. Luciferase activities were measured using the Dual-Glo luciferase assay kit (Gibco Life Technologies, Gaithersburg, MD, USA) as described previously by us [43,45]. Each experiment was performed in triplicate and repeated at least three times. Details of cell culture and luciferase assays were described in S1 Text.

### Real-time PCR analysis

The expression levels of *PITX2c*, *ZFHX3*, *NPPA*, *CAV1*, *NKX2*.*5*, *TBX5*, *KCNQ1*, and *SCN1B* were measured using real-time RT-PCR analysis with SYBR green I mix as described by us previously [36,44] and described in detail in S1 Text. Primers for real-time RT- PCR analysis are listed in S6 Table.

### Western blot analysis

Western blot analysis was carried out as described by us previously [43,44] and described in detail in S1 Text.

### Statistical analysis

The genotyping data for all SNPs are included in S8–S13 Tables. The genotyping data from the control group for each SNP were first tested for the Hardy-Weinberg equilibrium using PLINK1.06 (http://pngu.mgh.harvard.edu). If a *P* value was >0.01, the genotyping data were considered to be in the Hardy-Weinberg equilibrium. Genotypic frequencies in controls were all in Hardy-Weinberg equilibrium (*P*>0.01). For case-control association analysis, we used Pearson’s 2×2 and 2×3 contingency table χ^2^ tests as implemented in PLINK1.06 (http://pngu.mgh.harvard.edu) to compute the *P* values for allelic and genotypic associations, respectively. The same PLINK1.06 program was used to estimate the odds ratio (OR) and 95% confidence interval (CI) for each association. In order to exclude confounding factors, multivariable logistic regression analysis was performed using SPSS 17.0 to adjust for gender and age.

For analysis of gene-gene interaction, SNP rs2106261 in *ZFHX3* or SNP rs2200733 at the *PITX2c* locus each has two alleles (G vs. A for rs2106261; C vs. T for rs2200733). The two SNPs together generate nine different genotypes. We defined the homozygous, non-risk (or protective) two-locus genotype GGCC as the reference group, and then estimated the OR of AF for each of the other eight two-locus genotypes GGCT, GGTT, AGCC, AGCT, AGTT, AACC, AACT, and AATT in relation to the reference genotype. The Pearson’s 2×2 contingency table χ^2^ test was used to compute the nominal *P* values, ORs, and 95% CIs for each genotypic association using PLINK1.06. The Breslow-Day test was carried out to test whether the ORs between two different genotypes showed a statistically significant difference.

Gene-gene interaction was also measured by a relative excess risk due to interaction (RERI) analysis [19]. The RERI analysis analyzes was suggested to be more meaningful for disease prevention and intervention in public health [46], and advocated to be more biologically interpretable compared to that measured on the multiplicative scale [47]. A synergistic effect was defined as the extent of the combined effect of the exposures in excess of the sum of their individual effects [48]. We adopted a fundamental measure of RERI versus additive interaction to quantify the extent of synergistic effect in this study. The original form of RERI was defined as RERI = *RR*_11_-*RR*_10_-*RR*_01_+1, where subscript 11, 10 and 01 denote relative risks (RR) for doubly-exposed and individually-exposed to each risk factor when treating doubly-unexposed as a reference. When a RERI value equals to 0, it indicates a perfect additive model. Any significant deviation from 0 indicates a synergistic (+, positive values) or antagonistic (-, negative values) interaction. In a case-control study, RERI can be calculated by substituting ORs for RRs, yielding RERI = *OR*_11_-*OR*_10_-*OR*_01_+1. Although simply replacing RRs with ORs would induce an exaggeration problem for ORs [19,49], especially for a high prevalent disease, it is shown that RERI in terms of ORs is a good approximation of RERI in terms of RRs in a disease such as AF with a prevalence rate of 0.4%~0.8%) [49]. Under this circumstance, an OR is a good approximation of the RR. The statistical significance of RERI values in terms of ORs was addressed by the 95% confidence intervals based on the “MOVER” method, which utilizes the asymmetric intervals for ORs [19]. Since the SNPs are bi-allelic, it is of interest to explore if the interaction exists (1) when doubly-exposed to one copy of risk alleles (i.e. doubly heterozygous genotype) and (2) when doubly-exposed to two copies of risk alleles (i.e. double homozygous risk genotypes). In both scenarios, the doubly-unexposed is referred to as the homozygous non-risk genotype (e.g. GGCC). In addition, we tested the interaction when exposed one additional copy of risk alleles given being exposed to one copy of risk alleles. Note that the doubly-unexposed in this scenario is the doubly heterozygous genotype (e.g. AGCT). The *P* values were estimated by 10,000 times of bootstrap sampling. The *P* value of 0.05 or less than and the 95% CI of RERI through zero was considered to show statistical significance.

We also conducted a 4 degree of freedom test for genotypic interaction with logistic regression developed by Cordell and Clayton[20], which was implemented in the software INTERSNP[21] as Logistic Regression test #6. This model partitions the variance in AF risk into Additive and Dominant terms for each main effect, then into Additive by Additive, Additive by Dominant, Dominant by Additive and Dominant by Dominant terms. The test yielded ORs and 95% CI for each interaction term along with the global *P* values for the four terms. *P* values for individual terms were computed using Wald tests. We also used Logistic Regression test #5 in the INTERSNP program to test for additive interaction on a multiplicative ORs scale.

In molecular studies with quantitative data, a standard Student’s t-test was used to compare the means between two groups of variables. A *P* value of 0.05 or less was considered to show statistical significance.

## Supporting Information

S1 Fig Odds ratios (ORs) for each two-locus genotype for GWAS SNPs rs2106261 and rs2200733 involved in the pathogenesis of AF before adjustment for covariates. For two SNPs, there are a total of 9 genotypes. The wild type or non-risk GGCC genotype was used as the reference and ORs for other genotypes were estimated against the reference genotype using Pearson’s 2×2 contingency table χ^2^ tests using SPSS17.0. A. Analysis of ORs in the Discovery population. B. Analysis of ORs in the Replication I population. C. Analysis of ORs in the Replication II population. D. Analysis of ORs in the combined population with the Discovery, Replication I and Replication II cohorts. *P<0.01.
(TIF)

S1 Table Clinical characteristics of the Chinese Han populations used in the study. (XLSX)

S2 Table *P* values from Hardy-Weinberg Equilibrium tests in controls. (XLSX)

S3 Table Allelic association of rs2106261 and rs2200733 with AF in the Chinese Han population. (XLSX)

S4 Table Genotypic association of rs2106261 and rs2200733 with AF in the Chinese Han population. (XLSX)

S5 Table The Breslow-Day test of ORs between two different two-locus genotypes. (XLSX)

S6 Table Sequences for primers for PCR and real-time RT-PCR analyses. (XLSX)

S7 Table Sequences for siRNAs. (XLSX)

S8 Table Genotyping data of rs2106261 and rs2200733 in the discovery population. Gender: 1 = male, 2 = female; AF: 1 = control, 2 = case.
(XLSX)

S9 Table Genotyping data of rs2106261 and rs2200733 in the replication I population. Gender: 1 = male, 2 = female; AF: 1 = control, 2 = case.
(XLSX)

S10 Table Genotyping data of rs2106261 and rs2200733 in the replication II population. Gender: 1 = male, 2 = female; AF: 1 = control, 2 = case.
(XLSX)

S11 Table Genotyping data of rs2106261 and rs2200733 in the combined population. Gender: 1 = male, 2 = female; AF: 1 = control, 2 = case.
(XLSX)

S12 Table Genotyping data of rs2106261 and rs3807989. Gender: 1 = male, 2 = female; AF: 1 = control, 2 = case.
(XLS)

S13 Table Genotyping data of rs2200733 and rs3807989. Gender: 1 = male, 2 = female; AF: 1 = control, 2 = case.
(XLS)

S1 Text Study subjects and preparation of genomic DNA samples; genotyping of SNPs; prediction of potential miR-1 binding sites; plasmids, siRNAs, and microRNA mimics; real -time PCR analysis; western blot analysis; dual luciferase reporter assays. (DOC)

## References

[1] Go AS, Hylek EM, Phillips KA, Chang Y, Henault LE, et al (2001) Prevalence of diagnosed atrial fibrillation in adults: national implications for rhythm management and stroke prevention: the AnTicoagulation and Risk Factors in Atrial Fibrillation (ATRIA) Study. JAMA 285: 2370–2375. 11343485 10.1001/jama.285.18.2370

[2] Hu D, Sun Y (2008) Epidemiology, risk factors for stroke, and management of atrial fibrillation in China. J Am Coll Cardiol 52: 865–868. 10.1016/j.jacc.2008.05.042 18755352

[3] Fujii H, Kim JI, Yoshiya K, Nishi S, Fukagawa M (2011) Clinical characteristics and cardiovascular outcomes of hemodialysis patients with atrial fibrillation: a prospective follow-up study. Am J Nephrol 34: 126–134. 10.1159/000329118 21720157

[4] Christophersen IE, Ravn LS, Budtz-Joergensen E, Skytthe A, Haunsoe S, et al (2009) Familial aggregation of atrial fibrillation: a study in Danish twins. Circ Arrhythm Electrophysiol 2: 378–383. 10.1161/CIRCEP.108.786665 19808493 PMC2760022

[5] Gudbjartsson DF, Arnar DO, Helgadottir A, Gretarsdottir S, Holm H, et al (2007) Variants conferring risk of atrial fibrillation on chromosome 4q25. Nature 448: 353–357. 17603472 10.1038/nature06007

[6] Shi L, Li C, Wang C, Xia Y, Wu G, et al (2009) Assessment of association of rs2200733 on chromosome 4q25 with atrial fibrillation and ischemic stroke in a Chinese Han population. Hum Genet 126: 843–849. 10.1007/s00439-009-0737-3 19707791

[7] Gudbjartsson DF, Holm H, Gretarsdottir S, Thorleifsson G, Walters GB, et al (2009) A sequence variant in ZFHX3 on 16q22 associates with atrial fibrillation and ischemic stroke. Nat Genet 41: 876–878. 10.1038/ng.417 19597491 PMC2740741

[8] Benjamin EJ, Rice KM, Arking DE, Pfeufer A, van Noord C, et al (2009) Variants in ZFHX3 are associated with atrial fibrillation in individuals of European ancestry. Nat Genet 41: 879–881. 10.1038/ng.416 19597492 PMC2761746

[9] Li C, Wang F, Yang Y, Fu F, Xu C, et al (2011) Significant association of SNP rs2106261 in the ZFHX3 gene with atrial fibrillation in a Chinese Han GeneID population. Hum Genet 129: 239–246. 10.1007/s00439-010-0912-6 21107608 PMC5069458

[10] Ellinor PT, Lunetta KL, Glazer NL, Pfeufer A, Alonso A, et al (2010) Common variants in KCNN3 are associated with lone atrial fibrillation. Nat Genet 42: 240–244. 10.1038/ng.537 20173747 PMC2871387

[11] Ellinor PT, Lunetta KL, Albert CM, Glazer NL, Ritchie MD, et al (2012) Meta-analysis identifies six new susceptibility loci for atrial fibrillation. Nat Genet 44: 670–675. 10.1038/ng.2261 22544366 PMC3366038

[12] Chen S, Wang C, Wang X, Xu C, Wu M, et al (2015) Significant Association Between CAV1 Variant rs3807989 on 7p31 and Atrial Fibrillation in a Chinese Han Population. J Am Heart Assoc 4. 10.1161/JAHA.115.001980 PMC4599427 25953654

[13] Wang J, Klysik E, Sood S, Johnson RL, Wehrens XH, et al (2010) Pitx2 prevents susceptibility to atrial arrhythmias by inhibiting left-sided pacemaker specification. Proc Natl Acad Sci U S A 107: 9753–9758. 10.1073/pnas.0912585107 20457925 PMC2906838

[14] Kirchhof P, Kahr PC, Kaese S, Piccini I, Vokshi I, et al (2011) PITX2c is expressed in the adult left atrium, and reducing Pitx2c expression promotes atrial fibrillation inducibility and complex changes in gene expression. Circ Cardiovasc Genet 4: 123–133. 10.1161/CIRCGENETICS.110.958058 21282332

[15] Chinchilla A, Daimi H, Lozano-Velasco E, Dominguez JN, Caballero R, et al (2011) PITX2 insufficiency leads to atrial electrical and structural remodeling linked to arrhythmogenesis. Circ Cardiovasc Genet 4: 269–279. 10.1161/CIRCGENETICS.110.958116 21511879

[16] Ganga M, Espinoza HM, Cox CJ, Morton L, Hjalt TA, et al (2003) PITX2 isoform-specific regulation of atrial natriuretic factor expression: synergism and repression with Nkx2.5. J Biol Chem 278: 22437–22445. 12692125 10.1074/jbc.M210163200

[17] Hilton T, Gross MK, Kioussi C (2010) Pitx2-dependent occupancy by histone deacetylases is associated with T-box gene regulation in mammalian abdominal tissue. J Biol Chem 285: 11129–11142. 10.1074/jbc.M109.087429 20129917 PMC2856990

[18] Tao Y, Zhang M, Li L, Bai Y, Zhou Y, et al (2014) Pitx2, an atrial fibrillation predisposition gene, directly regulates ion transport and intercalated disc genes. Circ Cardiovasc Genet 7: 23–32. 10.1161/CIRCGENETICS.113.000259 24395921 PMC4013500

[19] Zou GY (2008) On the estimation of additive interaction by use of the four-by-two table and beyond. Am J Epidemiol 168: 212–224. 10.1093/aje/kwn104 18511428

[20] Cordell HJ, Clayton DG (2002) A unified stepwise regression procedure for evaluating the relative effects of polymorphisms within a gene using case/control or family data: application to HLA in type 1 diabetes. Am J Hum Genet 70: 124–141. 11719900 10.1086/338007 PMC384883

[21] Herold C, Steffens M, Brockschmidt FF, Baur MP, Becker T (2009) INTERSNP: genome-wide interaction analysis guided by a priori information. Bioinformatics 25: 3275–3281. 10.1093/bioinformatics/btp596 19837719

[22] Ritchie MD, Rowan S, Kucera G, Stubblefield T, Blair M, et al (2012) Chromosome 4q25 variants are genetic modifiers of rare ion channel mutations associated with familial atrial fibrillation. J Am Coll Cardiol 60: 1173–1181. 10.1016/j.jacc.2012.04.030 22818067 PMC3448817

[23] Lubitz SA, Lunetta KL, Lin H, Arking DE, Trompet S, et al (2014) Novel genetic markers associate with atrial fibrillation risk in Europeans and Japanese. J Am Coll Cardiol 63: 1200–1210. 10.1016/j.jacc.2013.12.015 24486271 PMC4009240

[24] Franco D, Campione M (2003) The role of Pitx2 during cardiac development. Linking left-right signaling and congenital heart diseases. Trends Cardiovasc Med 13: 157–163. 12732450 10.1016/s1050-1738(03)00039-2

[25] Faucourt M, Houliston E, Besnardeau L, Kimelman D, Lepage T (2001) The pitx2 homeobox protein is required early for endoderm formation and nodal signaling. Dev Biol 229: 287–306. 11203696 10.1006/dbio.2000.9950

[26] Mommersteeg MT, Hoogaars WM, Prall OW, de Gier-de Vries C, Wiese C, et al (2007) Molecular pathway for the localized formation of the sinoatrial node. Circ Res 100: 354–362. 17234970 10.1161/01.RES.0000258019.74591.b3

[27] Yasuda H, Mizuno A, Tamaoki T, Morinaga T (1994) ATBF1, a multiple-homeodomain zinc finger protein, selectively down-regulates AT-rich elements of the human alpha-fetoprotein gene. Mol Cell Biol 14: 1395–1401. 7507206 10.1128/mcb.14.2.1395-1401.1994 PMC358494

[28] Morinaga T, Yasuda H, Hashimoto T, Higashio K, Tamaoki T (1991) A human alpha-fetoprotein enhancer-binding protein, ATBF1, contains four homeodomains and seventeen zinc fingers. Mol Cell Biol 11: 6041–6049. 1719379 10.1128/mcb.11.12.6041-6049.1991 PMC361769

[29] Berry FB, Miura Y, Mihara K, Kaspar P, Sakata N, et al (2001) Positive and negative regulation of myogenic differentiation of C2C12 cells by isoforms of the multiple homeodomain zinc finger transcription factor ATBF1. J Biol Chem 276: 25057–25065. 11312261 10.1074/jbc.M010378200

[30] Jung CG, Kim HJ, Kawaguchi M, Khanna KK, Hida H, et al (2005) Homeotic factor ATBF1 induces the cell cycle arrest associated with neuronal differentiation. Development 132: 5137–5145. 16251211 10.1242/dev.02098

[31] Ido A, Miura Y, Watanabe M, Sakai M, Inoue Y, et al (1996) Cloning of the cDNA encoding the mouse ATBF1 transcription factor. Gene 168: 227–231. 8654949 10.1016/0378-1119(95)00740-7

[32] Wang F, Xu CQ, He Q, Cai JP, Li XC, et al (2011) Genome-wide association identifies a susceptibility locus for coronary artery disease in the Chinese Han population. Nat Genet 43: 345–349. 10.1038/ng.783 21378986

[33] Xu C, Wang F, Wang B, Li X, Li C, et al (2010) Minor allele C of chromosome 1p32 single nucleotide polymorphism rs11206510 confers risk of ischemic stroke in the Chinese Han population. Stroke 41: 1587–1592. 10.1161/STROKEAHA.110.583096 20576952

[34] Cheng X, Shi L, Nie S, Wang F, Li X, et al (2011) The same chromosome 9p21.3 locus is associated with type 2 diabetes and coronary artery disease in a Chinese Han population. Diabetes 60: 680–684. 10.2337/db10-0185 21270277 PMC3028370

[35] Li X, Huang Y, Yin D, Wang D, Xu C, et al (2013) Meta-analysis identifies robust association between SNP rs17465637 in MIA3 on chromosome 1q41 and coronary artery disease. Atherosclerosis 231: 136–140. 10.1016/j.atherosclerosis.2013.08.031 24125424

[36] Xiong X, Xu C, Zhang Y, Li X, Wang B, et al (2014) BRG1 variant rs1122608 on chromosome 19p13.2 confers protection against stroke and regulates expression of pre-mRNA-splicing factor SFRS3. Hum Genet 133: 499–508. 10.1007/s00439-013-1389-x 24190014 PMC3988217

[37] Bai Y, Nie S, Jiang G, Zhou Y, Zhou M, et al (2014) Regulation of CARD8 expression by ANRIL and association of CARD8 single nucleotide polymorphism rs2043211 (p.C10X) with ischemic stroke. Stroke 45: 383–388. 10.1161/STROKEAHA.113.003393 24385277 PMC3962686

[38] Tu X, Nie S, Liao Y, Zhang H, Fan Q, et al (2013) The IL-33-ST2L pathway is associated with coronary artery disease in a Chinese Han population. Am J Hum Genet 93: 652–660. 10.1016/j.ajhg.2013.08.009 24075188 PMC3791271

[39] Ren X, Xu C, Zhan C, Yang Y, Shi L, et al (2010) Identification of NPPA variants associated with atrial fibrillation in a Chinese GeneID population. Clin Chim Acta 411: 481–485. 10.1016/j.cca.2009.12.019 20064500

[40] Fuster V, Ryden LE, Cannom DS, Crijns HJ, Curtis AB, et al (2006) ACC/AHA/ESC 2006 Guidelines for the Management of Patients with Atrial Fibrillation: a report of the American College of Cardiology/American Heart Association Task Force on Practice Guidelines and the European Society of Cardiology Committee for Practice Guidelines (Writing Committee to Revise the 2001 Guidelines for the Management of Patients With Atrial Fibrillation): developed in collaboration with the European Heart Rhythm Association and the Heart Rhythm Society. Circulation 114: e257–354. 16908781 10.1161/CIRCULATIONAHA.106.177292

[41] Oberti C, Wang L, Li L, Dong J, Rao S, et al (2004) Genome-wide linkage scan identifies a novel genetic locus on chromosome 5p13 for neonatal atrial fibrillation associated with sudden death and variable cardiomyopathy. Circulation 110: 3753–3759. 15596564 10.1161/01.CIR.0000150333.87176.C7 PMC1618875

[42] Zhang X, Chen S, Yoo S, Chakrabarti S, Zhang T, et al (2008) Mutation in nuclear pore component NUP155 leads to atrial fibrillation and early sudden cardiac death. Cell 135: 1017–1027. 10.1016/j.cell.2008.10.022 19070573

[43] Zhou B, Ma R, Si W, Li S, Xu Y, et al (2013) MicroRNA-503 targets FGF2 and VEGFA and inhibits tumor angiogenesis and growth. Cancer Lett 333: 159–169. 10.1016/j.canlet.2013.01.028 23352645

[44] Xu Y, Zhou M, Wang J, Zhao Y, Li S, et al (2014) Role of microRNA-27a in down-regulation of angiogenic factor AGGF1 under hypoxia associated with high-grade bladder urothelial carcinoma. Biochim Biophys Acta 1842: 712–725. 10.1016/j.bbadis.2014.01.007 24462738

[45] Fan C, Liu M, Wang Q (2003) Functional analysis of TBX5 missense mutations associated with Holt-Oram syndrome. J Biol Chem 278: 8780–8785. 12499378 10.1074/jbc.M208120200 PMC1579789

[46] Rothman KJ (1976) The estimation of synergy or antagonism. Am J Epidemiol 103: 506–511. 1274952 10.1093/oxfordjournals.aje.a112252

[47] Greenland S, Poole C (1988) Invariants and noninvariants in the concept of interdependent effects. Scand J Work Environ Health 14: 125–129. 3387960 10.5271/sjweh.1945

[48] Wang X, Elston RC, Zhu X (2010) The meaning of interaction. Hum Hered 70: 269–277. 10.1159/000321967 21150212 PMC3025890

[49] Kalilani L, Atashili J (2006) Measuring additive interaction using odds ratios. Epidemiol Perspect Innov 3: 5 16620385 10.1186/1742-5573-3-5 PMC1475799

