# Supplementary figures and images for: Molecular Basis of Gene-Gene Interaction: Cyclic Cross-Regulation of Gene Expression and Post-GWAS Gene-Gene Interaction Involved in Atrial Fibrillation
Source: PLoS Genet. 2015 Aug 12;11(8):e1005393. doi: 10.1371/journal.pgen.1005393 (PMC4534423; doi:10.1371/journal.pgen.1005393)

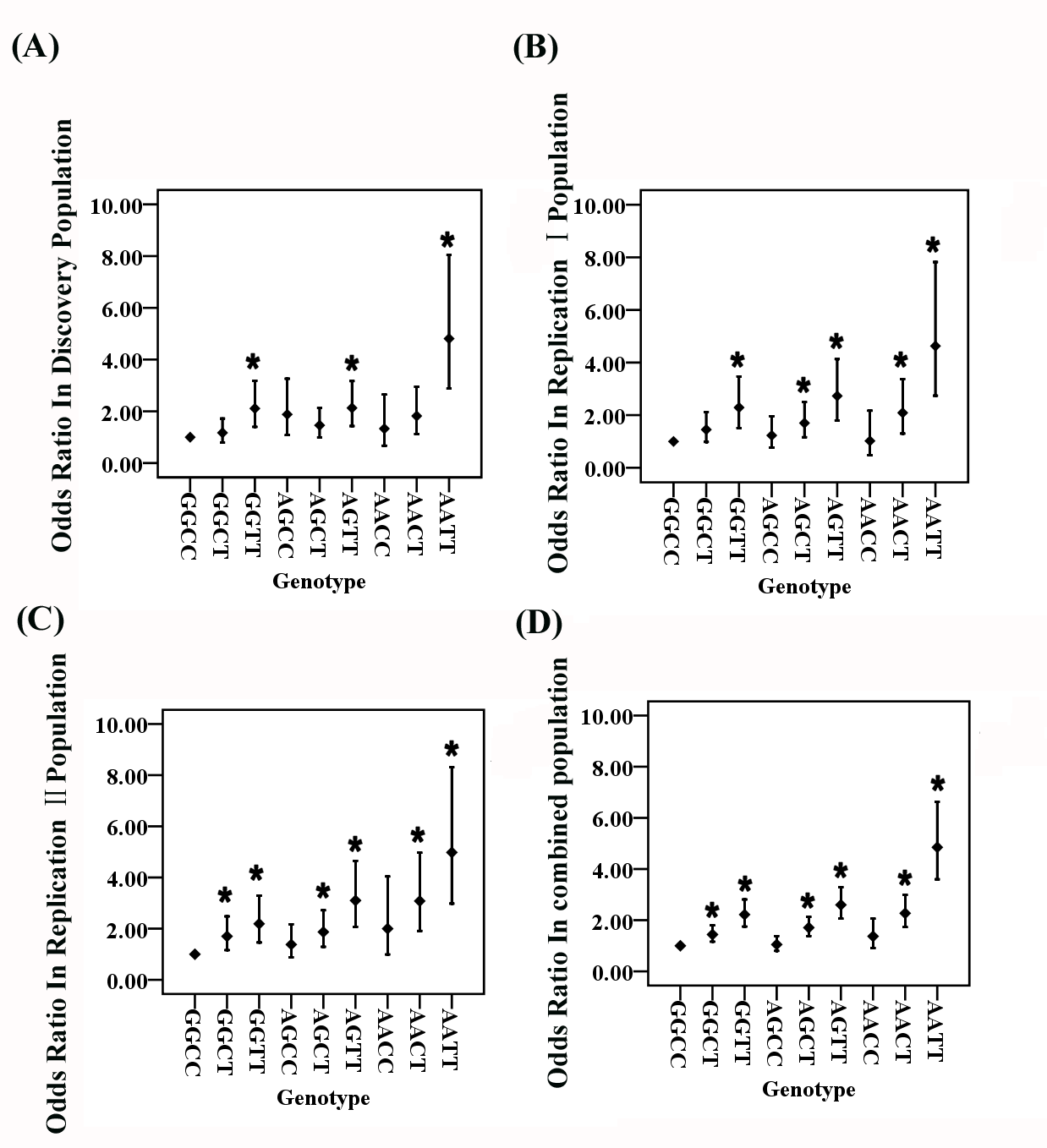

Supplement: S1 Fig — For two SNPs, there are a total of 9 genotypes. The wild type or non-risk GGCC genotype was used as the reference and ORs for other genotypes were estimated against the reference genotype using Pearson’s 2×2 contingency table χ2 tests using SPSS17.0. A. Analysis of ORs in the Discovery population. B. Analysis of ORs in the Replication I population. C. Analysis of ORs in the Replication II population. D. Analysis of ORs in the combined population with the Discovery, Replication I and Replication II cohorts. *P<0.01. (TIF) [file pgen.1005393.s001.tif]
