# Supplementary material for: Molecular Basis of Gene-Gene Interaction: Cyclic Cross-Regulation of Gene Expression and Post-GWAS Gene-Gene Interaction Involved in Atrial Fibrillation
Source: PLoS Genet. 2015 Aug 12;11(8):e1005393. doi: 10.1371/journal.pgen.1005393 (PMC4534423; doi:10.1371/journal.pgen.1005393)
Supplement: S1 Text — (DOC) [file pgen.1005393.s015.doc]

# Supporting Information

# Molecular Basis of Gene-Gene Interaction: Cyclic Cross-Regulation of Gene Expression and Post-GWAS Gene-Gene Interaction Involved in Atrial Fibrillation

**Study subjects and preparation of genomic DNA samples**

The subjects involved in the present study include AF patients and non-AF controls selected from the GeneID database. GeneID is a large ongoing cardiovascular database that aims to identify major genes related to many cardiovascular diseases, including AF, ventricular tachycardia (VT), coronary artery disease (CAD) and myocardial infarction (MI), stroke, hypertension, congenital heart disease, cardiomyopathies and heart failure, and related complex disease traits . The GeneID database has enrolled more than 80,000 study subjects to date from more than ten large hospitals in several major cities and provinces in China. All study subjects are of Han ethnic origin based on self-description.

The study was approved by the Ethics Committee of Huazhong University of Science and Technology and the Ethics Committees from local hospitals, and consistent with the guideline in the Declaration of Helsinki. Written informed consent was obtained from the participants.

The diagnosis of AF was made by multiple experienced cardiologists and cardiac electrophysiologists using data from 12-lead surface electrocardiograms (ECGs) or Holter recordings. The ECG characteristics of AF include the absence of P waves, the presence of rapid oscillations or fibrillatory waves (F waves), and irregular R-R intervals . The controls are healthy individuals who do not have AF at the time of physical examinations or from medical records.

Human genomic DNA samples were extracted form whole blood samples using the Wizard® Genomic DNA Purification Kit (Promega Corporation, Madison, WI, USA) according to the manufacturer’s protocol.

**Genotyping of SNPs**

SNP genotyping was carried out using a Rotor-Gene 6000 High-Resolution Melt (HRM) system (Corbett Life Science, Concorde, NSW, Australia) as described previously by us . PCR primers for genotyping are in Table S6. PCR was performed in a 25 μl of standard PCR volume containing 1 μl of LC green dye, 5 pmol of each primer, 25 ng of genomic DNA, 2.5 μl of 10×PCR buffer with 1.5 mM MgCl_2_, 5 mmol deoxynucleotide triphosphates, and 1 unit of Taq polymerase. The PCR program was 94°C for 5 min, 40 cycles of 10 s at 94°C, 10 s at the annealing temperature, and 10 s at 72°C, and 72°C for 10 min. Two positive DNA samples with known genotypes were included in each run of genotyping. We also included water alone (i.e. no genomic DNA) as negative control.

The validity of SNP genotyping was validated by direst DNA sequencing analysis of 26 randomly selected controls and 26 randomly selected patients in each population.

**Prediction of potential miR-1 binding sites**

The target binding sites of *miR-1* were predicted by searching two databases: DIANA microT-CDS V5.0 (http://diana.imis.athena-innovation.gr/DianaTools/) [and microRNA.org-Target and Expression(http://www.microrna.org/microrna/home.do). *ZFHX3*](http://diana.cslab.ece.ntua.gr/micro-CDS/?r=search).ZFHX3) was predicted to be a downstream target gene of *miR-1* and contain two *miR-1* binding sites at the 3’-untraslated region (UTR).

**Plasmids, siRNAs, and microRNA mimics**

Total RNA from human cardiac tissue samples was converted to cDNA by reverse transcription, and used to amplify the *PITX2c* cDNA using PCR analysis. The PCR product (the longest *PITX2* transcript 005) was digested with *Sal I* and *BamH I* restriction enzymes and sub-cloned into the multiple cloning site of vector p3×FLAG-CMV-7.1-8 cut with the same enzymes. This expression construct for *PITX2c* was referred to as p3×FLAG-PITX2c.

The expression plasmid for *ZFHX3* (also referred to as *HH-ATBF1*) was kindly provided by Dr. Jin-Tang Dong at Emory University School of Medicine .

For the construction of a *PITX2c* promoter luciferase reporter (PITX2c-PGL3), a genomic region from −1,500 to +1 bp upstream from the transcription start site of the *PITX2c* promoter were PCR-amplified from human genomic DNA and separately cloned into the pGL3-Basic vector using *Mlu I* and *Xho I* restriction enzymes, resulting in PITX2c-PGL3.

The 3’-UTR region of *ZFHX3* with each predicted *miR-1* target binding sequence was amplified by PCR analysis using human genomic DNA. The PCR product was digested with *Spe I* and *Sac I* restriction enzymes and sub-cloned into the multiple cloning site of vector pMIR-REPORT luciferase (Applied Biosystems, Foster City, CA, USA) cut with the same enzymes. The resulting constructs were referred to as pMIR-ZFHX3-3’UTR-1 and pMIR-ZFHX3-3’UTR-2, respectively. In each construct, the 3’-UTR fragment was inserted downstream of the firefly *luciferase* coding region.

The siRNAs specifically targeting *PITX2c*, *ZFHX3* or *CAV1* were synthesized by Su Zhou Gene Pharma Co. The sequences of siRNAs are listed in Table S7.

*MiR-1* mimics and control miR mimics were purchased from Guangzhou RiboBio.

**Real -time PCR analysis**

HCT116 cells were cultured in 12-well plates and transfected with 1.6 ug of p3×FLAG-PITX2c, HA-ATBF1, and empty vector pcDNA3.1(+) or pEGFP-N1 . For RNA interference experiments, cells were transfected with 40 pmol specific siRNA or a negative control scramble siRNA. For *miR1* mimics, cells were transfected with miR-1 mimics or negative control mimics. The transfection was performed using lipofectamine 2000 and the Opti-MEM I reduced serum medium (Gibco Life Technologies, Gaithersburg, MD, USA) according to the manufacturer’s the protocol. Forty-eight hours after transfection, cells were harvested and used for isolation of total RNA using RNAiso plus. Total RNA was converted into cDNA by reverse transcription with the First-Strand cDNA Synthesis kit (Invitrogen, Carlsbad, CA, USA) using random primers. Real-time PCR analysis was carried out with a FastStart Universal SYBR Green Master kit (Roche Applied Science, Mannheim, Germany) in a 10 μl reaction volume on an ABI 7900 Genome Analyzer System (5 ul SYBR green I mix, 200 mM forward and reverse primers, 1ul cDNA template). The PCR profile was 94^0^C for 5 min, and 40 cycles of 94°C for 10 s and 60°C for 15 s. Primer for real-time PCR analysis of *PITX2c*, *ZFHX3*, *NPPA*, *CAV1* , *NKX2.5*, *TBX5*, *KCNQ1*, *SCN1B* and control *GAPDH* are in Table S6. Data analysis was performed using the 2-^△△^Ct method as described previously by us .

**Western blot analysis**

Transfected cells were harvested and used for preparation of protein extracts. Cells were lysed in lysis buffer (20 mM Tris–HCl, pH 7.4, 150 mM NaCl, 1 mM EDTA, 1% NP-40, proteinase inhibitor cocktail), and total protein extracts were used for Western blot analysis as described add many references form our lab . An equal amount of protein extracts (50 μg) were separated by 10% SDS–PAGE and transferred to a PVDF membrane. The membrane was probed with a primary antibody for 2 h at room temperature. The following antibodies were used for Western blotting: a rabbit polyclonal antibody against ZFHX3 (1:1 000, Abcam, USA), a rabbit polyclonal antibody to PITX2c (1:500, Abcam, USA), a rabbit polyclonal antibody to CAV1 (1:500, Abcam, USA) and a mouse monoclonal antibody for beta-actin (1:1 000, Abcam, USA). The membranes were then probed using either a goat anti-rabbit or a goat anti-mouse HRP-conjugated secondary antibody (1:20,000, Sigma Aldrich, Castle Hill, NSW, Australia). Signals were then detected by incubation with a SuperSignal West Pico Chemiluminescent Substrate (Pierce Chemical Co., Rockford, Illinois, USA), imaged using a ChemiDoc XRS (Bio-Rad Laboratories, Richmond, CA), and quantified using Quantity One software (Bio-Rad Laboratories, Richmond, CA) as described previously by us .

**Dual luciferase reporter assays**

HCT116 cells were cultured in 24-well plates and transfected with 200 ng of either pMIR-ZFHX3-3’UTR-1, pMIR-ZFHX3-3’UTR-2 or the empty pMIR-Report plasmid in combination with 100 nM of *miR-1* or non-target miRNA control mimics, along with 20 ng of pRL-TK vector containing *Renilla luciferase* (Promega, Madison, WI, USA) using lipofectamine 2000 and the Opti-MEM I reduced serum medium (Gibco Life Technologies, Gaithersburg, MD, USA) according to the manufacturer’s the protocol. Similarly, cells were transfected with 200 ng of PITX2c-PGL3, siRNA specific for *ZFHX3* and 20 ng of pRL-TK vector containing Renilla luciferase using lipofectamine 2000.

Forty-eight hours after transfection, cells were harvested, lysed using 1× passive lysis buffer (Promega, Madison, WI, USA) and used for luciferase assays. Firefly and Renilla luciferase activities were measured using the Dual-Glo luciferase assay kit (Gibco Life Technologies, Gaithersburg, MD, USA) as described previously by us . Each experiment was performed in triplicate and repeated at least three times.

**References**

1. Shi L, Li C, Wang C, Xia Y, Wu G, et al. (2009) Assessment of association of rs2200733 on chromosome 4q25 with atrial fibrillation and ischemic stroke in a Chinese Han population. Hum Genet 126: 843-849.

2. Xu C, Wang F, Wang B, Li X, Li C, et al. (2010) Minor allele C of chromosome 1p32 single nucleotide polymorphism rs11206510 confers risk of ischemic stroke in the Chinese Han population. Stroke 41: 1587-1592.

3. Li C, Wang F, Yang Y, Fu F, Xu C, et al. (2011) Significant association of SNP rs2106261 in the ZFHX3 gene with atrial fibrillation in a Chinese Han GeneID population. Hum Genet 129: 239-246.

4. Wang F, Xu CQ, He Q, Cai JP, Li XC, et al. (2011) Genome-wide association identifies a susceptibility locus for coronary artery disease in the Chinese Han population. Nat Genet 43: 345-349.

5. Ren X, Xu C, Zhan C, Yang Y, Shi L, et al. (2010) Identification of NPPA variants associated with atrial fibrillation in a Chinese GeneID population. Clin Chim Acta 411: 481-485.

6. Li X, Huang Y, Yin D, Wang D, Xu C, et al. (2013) Meta-analysis identifies robust association between SNP rs17465637 in MIA3 on chromosome 1q41 and coronary artery disease. Atherosclerosis 231: 136-140.

7. Bai Y, Nie S, Jiang G, Zhou Y, Zhou M, et al. (2014) Regulation of CARD8 expression by ANRIL and association of CARD8 single nucleotide polymorphism rs2043211 (p.C10X) with ischemic stroke. Stroke 45: 383-388.

8. Xiong X, Xu C, Zhang Y, Li X, Wang B, et al. (2014) BRG1 variant rs1122608 on chromosome 19p13.2 confers protection against stroke and regulates expression of pre-mRNA-splicing factor SFRS3. Hum Genet 133: 499-508.

9. Tu X, Nie S, Liao Y, Zhang H, Fan Q, et al. (2013) The IL-33-ST2L pathway is associated with coronary artery disease in a Chinese Han population. Am J Hum Genet 93: 652-660.

10. Fuster V, Ryden LE, Cannom DS, Crijns HJ, Curtis AB, et al. (2006) ACC/AHA/ESC 2006 Guidelines for the Management of Patients with Atrial Fibrillation: a report of the American College of Cardiology/American Heart Association Task Force on Practice Guidelines and the European Society of Cardiology Committee for Practice Guidelines (Writing Committee to Revise the 2001 Guidelines for the Management of Patients With Atrial Fibrillation): developed in collaboration with the European Heart Rhythm Association and the Heart Rhythm Society. Circulation 114: e257-354.

11. Oberti C, Wang L, Li L, Dong J, Rao S, et al. (2004) Genome-wide linkage scan identifies a novel genetic locus on chromosome 5p13 for neonatal atrial fibrillation associated with sudden death and variable cardiomyopathy. Circulation 110: 3753-3759.

12. Zhang X, Chen S, Yoo S, Chakrabarti S, Zhang T, et al. (2008) Mutation in nuclear pore component NUP155 leads to atrial fibrillation and early sudden cardiac death. Cell 135: 1017-1027.

13. Dong XY, Sun X, Guo P, Li Q, Sasahara M, et al. (2010) ATBF1 inhibits estrogen receptor (ER) function by selectively competing with AIB1 for binding to the ER in ER-positive breast cancer cells. J Biol Chem 285: 32801-32809.

14. Zhou B, Ma R, Si W, Li S, Xu Y, et al. (2013) MicroRNA-503 targets FGF2 and VEGFA and inhibits tumor angiogenesis and growth. Cancer Lett 333: 159-169.

15. Xu Y, Zhou M, Wang J, Zhao Y, Li S, et al. (2014) Role of microRNA-27a in down-regulation of angiogenic factor AGGF1 under hypoxia associated with high-grade bladder urothelial carcinoma. Biochim Biophys Acta 1842: 712-725.

16. Su Z, Si W, Li L, Zhou B, Li X, et al. (2014) MiR-144 regulates hematopoiesis and vascular development by targeting meis1 during zebrafish development. Int J Biochem Cell Biol 49: 53-63.

17. Chen D, Li L, Tu X, Yin Z, Wang Q (2013) Functional characterization of Klippel-Trenaunay syndrome gene AGGF1 identifies a novel angiogenic signaling pathway for specification of vein differentiation and angiogenesis during embryogenesis. Hum Mol Genet 22: 963-976.

18. Li L, Chen D, Li J, Wang X, Wang N, et al. (2014) Aggf1 acts at the top of the genetic regulatory hierarchy in specification of hemangioblasts in zebrafish. Blood 123: 501-508.

19. Fan C, Liu M, Wang Q (2003) Functional analysis of TBX5 missense mutations associated with Holt-Oram syndrome. J Biol Chem 278: 8780-8785.
